# Target proteins reprogrammed by As and As + Si treatments in *Solanum lycopersicum* L. fruit

**DOI:** 10.1186/s12870-017-1168-2

**Published:** 2017-11-21

**Authors:** Marta Marmiroli, Francesca Mussi, Davide Imperiale, Nelson Marmiroli

**Affiliations:** 1 Department of Chemistry, Life Sciences and Environmental Sustainability, University of Parma, Parco Area delle Scienze 33/A, 43124 Parma, Italy; 2 Department of Chemistry, Life Sciences and Environmental Sustainability, University of Parma, Parco Area delle Scienze 11/A, 43124 Parma, Italy

**Keywords:** Proteomics, Oxidative stress, Fruit development, Environmental toxin, Beneficial element

## Abstract

**Background:**

Arsenic is an important contaminant of many arable soils worldwide, while silicon, one of the most abundant elements in the earth’s crust, interacts with As in the context of plant metabolism. As toxicity results largely from its stimulation of reactive oxygen species, and it is believed that Si can mitigate this process through reduction of the level of oxidative stress. Experiments targeting the proteomic impact of exposure to As and Si have to date largely focused on analyses of root, shoot and seed of a range of mainly non-solanaceous species, thus it remains unclear whether oxidative stress is the most important manifestation of As toxicity in *Solanum lycopersicum* fruit which during ripening go through drastic physiological and molecular readjustments. The role of Si also needs to be re-evaluated.

**Results:**

A comparison was drawn between the proteomic responses to As and As + Si treatments of the fruit of two tomato cultivars (cvs. Aragon and Gladis) known to contrast for their ability to take up these elements and to translocate them into fruits. Treatments were applied at the beginning of the red ripening stage, and the fruit proteomes were captured after a 14 day period of exposure. For each cultivar, a set of differentially abundant fruit proteins (from non-treated and treated plants) were isolated by 2DGE and identified using mass spectrometry. In the fruit of cv. Aragon, the As treatment reprogrammed proteins largely involved in transcription regulation (growth- regulating factor 9-like), and cell structure (actin-51), while in the cv. Gladis, the majority of differentially expressed proteins were associated with protein ubiquitination and proteolysis (E3 ubiquitin protein, and hormones (1-aminocyclopropane 1-carboxylase).

**Conclusions:**

The present experiments were intended to establish whether Si supplementation can be used to reverse the proteomic disturbance induced by the As treatment; this reprogram was only partial and more effective in the fruit of cv. Gladis than in that of cv. Aragon. Proteins responsible for the protection of the fruits’ quality in the face of As-induced stress were identified. Moreover, supplementation with Si seemed to limit to a degree the accumulation of As in the tomato fruit of cv. Aragon.

**Electronic supplementary material:**

The online version of this article (10.1186/s12870-017-1168-2) contains supplementary material, which is available to authorized users.

## Background

The important crop plant tomato has also been adopted as the leading genetic model for solanaceous species [1, 2]. Over 75,000 accessions are maintained by various gene banks, and some 7000 cultivars are grown commercially around the world [3]. Two independent versions of the species’ genomic DNA sequence have been acquired (one from the inbred cultivar Heinz 1706 and the other from the close wild relative *Solanum pimpinellifolium* LA1589, http://solgenomics.net/).

The development of the tomato fruit proceeds through several major stages [4]: the early phase is characterized by rapid cell division, growth and endoreduplication [5], the mature green stage by extensive metabolic reorganization [6] and the final stages by the conversion of chloroplasts into chromoplasts, the accumulation of carotenoids and the degradation of chlorophyll [7, 8]. At the onset of the red ripening stage, starch is metabolized into glucose, fructose and various organic acids [2]. A proteomic scan of the fruit of eight tomato cultivars has defined over 1200 gel features, of which around a third varied in intensity during the ripening stage, while a comparison between six cultivars at the cell expansion and orange-red stages revealed over 1000 features [9, 10]. The chloroplast-to-chromoplast transition similarly involves large numbers of proteins [11].

Arsenic in the environment is of geochemical and anthropic origin, in the Earth’s crust, total As is estimated to be 4.01×10^16^ kg [12]. 1.715×10^7^ kg/year of As is released from lithosphere through terrestrial volcanic exhalations and eruptions. Industry, mining and agriculture release into the environment 80 Mt./year of As [13]. The As contaminates water bodies, drinking water, and contributes to crop contamination through irrigation [12, 14]. Fertilizers and pesticides release As in agricultural soil where some crop species, such as rice and other vegetables may take up and translocate As in their edible parts [15, 16].

Excessive levels of arsenic (As) can be toxic to many plant species: its presence induces oxidative stress, suppresses photosynthesis and disrupts metabolism by replacing the phosphate in ATP with the unstable form ADP-As [17–19]. While silicon (Si) is not generally considered to be essential for plant growth and development, its presence does reduce the uptake and root-to-shoot transport of certain heavy metal ions and metalloids, including As [20–22]. It is believed that Si application can reduce the level of oxidative stress experienced in plant tissue by bearing down on the production of reactive oxygen species (ROS), by enhancing the activity of various antioxidants, by adjusting the osmotic potential of the cell and by increasing photosynthetic activity [23–25]. The effect of both exogenously supplied As and Si on the plant proteome has been documented in several species [26, 27]. In rice, a strong interaction between the two elements has been demonstrated [23, 28, 29]. The growth and development of the tomato is strongly affected by both elements: for example, Si influences the amount of As translocated to the tomato shoot in a cultivar-dependent fashion [30–32]. To date, their proteomic effect in tomato has been determined in the root and shoot [33–35], but not as yet in the fruit. In a comparison drawn between the two rather closely related cultivars Aragon and Gladis, Marmiroli et al. [31] were able to demonstrate differences in the amount of As reaching the fruit following exposure of the plant to either As on its own or in combination with Si.

Fruit provides humans with a source of nutritional elements and beneficial molecules, in particular tomato berry constitutes one of the main staples of the healthy “Mediterranean diet” [36]. Since the early domestication, tomato plants have been selected for useful traits, among which increase in fruit size and, more recently, fruit shape variety and high sugars content [1].

All fruit metabolism is organized in networks in which proteins such as enzymes, transcription factors, and transporters play a crucial role. When the ripening process evolves in time, as well as in response to environmental stimuli this networking is reprogrammed [37–39].

Therefore environmental cues interact with the genotype causing alterations in fruit enzymes pool ultimately leading to conspicuous changes in fruit metabolites and organoleptic characteristics [37, 40, 41].

The purpose of the present experiments was to characterize the effect on the fruit proteome of exposure to As and As + Si in genetically similar, but materially different with respect to their response to these treatments, in particular, to the extent to which they accumulate As in their fruit [31].

The fruit proteome of the two cultivars responded to the As and As + Si treatments in distinct ways: in cv. Aragon fruit, Si exerted a more effective protection than in the fruit of cv. Gladis. Indeed in fruits treated with the As and As + Si the proteome reprogramming resulted in part overlapped with changes associated with the ripening process.

## Methods

### Reagents and standards

All reagents and standards were purchased from Sigma-Aldrich (St. Louis, MO, USA) unless stated otherwise.

### Plants growth conditions

The soil was a mixture of 20% silica, 40% sphagnum moss peat (Presto Durpes UAB, Vilnius, Lithuania) and 40% black peat and wood fiber (Ecomix, Vialca S.R.L., Uzzano, Italy), which was passed through a 5 mm sieve, sterilized by baking at 120 °C for 1 h, then held at 50 °C for around 72 h until a constant weight had been attained. Seedlings of cvs Aragon and Gladis were raised for 4 weeks under a 14 h photoperiod provided by 300 μmol m^−2^ s^−1^ metal halide lamps in cabinets providing a 23/16 °C day/night temperature regime and a relative humidity of 50%. They were then transplanted in 9 L pots and irrigated with 500 mL tap water (pH 7.5, EC 0.6-0.7 dS m^−1^) every 2 days. The soils’ electrical conductivity (EC) and pH were monitored following the EPA methods 9045D and 9050A. Once a week, each pot was fertilized by adding 200 mL of 2% *w*/*v* blood meal (Guaber S.R.L., Bologna, Italy). The plants were raised in a greenhouse providing a day/night temperature of 25-30/13-16 °C, with the natural light supplemented by 14 h per day of 300 μmol m^−2^ s^−1^ light provided by metal halide lamps. Throughout the experiment, the soils’ pH varied in the range 6.3-6.7, while their electrical conductivity lay between 4.2 and 4.6 dS m^−1^.

### Experimental design and treatments

The As and As + Si treatments were initiated during the ripening of the first fruit (about 100 days after sowing). For the As treatment, each pot was watered with 2 L of 5 mg L^−1^ NaAsO_2_, for the As + Si treatment, 2 L of 5 mg L^−1^ NaAsO_2_ combined with 2 mg L^−1^ CaSiO_3_, while a set of control plants (nt) received neither supplement. The fruit was sampled both before the treatments commenced (t0) and 14 days later (t14d), selecting fruit of a comparable size and developmental stage, according to days after flower anthesis and color, were harvested [42].

Treatments concentrations and time were chosen according to previous work by the Authors [31] and other literature on tomato fruit proteomics [9, 43].

All sampled fruits were positioned between 6th and 8th leaf nodes. Each cultivar/treatment type/sampling time combination was represented by four plants, with at least three fruits per treatment taken for analysis. After harvest, the fruits were rinsed in deionized water and the replicates combined into single samples; pericarp and cuticle tissue was retained, while the placenta and seeds were discarded. The samples were snap frozen in liquid nitrogen and stored at −80 °C until use.

### Determination of As content

To measure the As content of the fruit samples, a 300 mg aliquot of powdered material was digested in 15 mL 14.6 M HNO_3_ for 60 min at 165 °C following Marmiroli et al. [31]. The resulting solution was subsequently diluted to 6.7 M HNO_3_ using distilled water. The absorbance of each sample was read using an AA240FS device (Agilent Technologies, Santa Clara, CA, USA) equipped with a Varian VGA 77 vapor generator assembly. The absorbance, captured at 189 nm, was converted into an As concentration based on a standard curve generated from a serial dilution of a 10,000 ppm standard solution. All analyses were performed in triplicate. The As concentration of the soil, tap water and blood meal, measured in the same way, lay consistently below the detection limit (BDL), defined as 0.001 μg g^−1^.

### Determination of Si content

Inductively coupled plasma optical emission spectrometry was employed to determine the fruits’ Si content following van der Vorm [44]. Fruits were dried, powdered, and a 300 mg aliquot of the powdered fruit material was ashed by holding at 550 °C for 3 h, and the ash suspended in 12.5 mL 0.08 M H_2_SO_4_ (Carlo Erba, Milan, Italy) plus 0.5 mL 23 M HF (Acros Organics, Geel, Belgium). The resulting suspension was shaken for 1 h, then held overnight. The supernatant was assayed using an Optima 7300 DV device (Perkin Elmer, Waltham, MA, USA). The instrument parameters were set as: power 1.4 kW; plasma gas flow rate 15 L min^−1^; nebulizer gas flow rate 0.78 L min^−1^; auxiliary gas flow rate 0.2 L min^−1^; sample flow rate 0.85 mL min^−1^; Si wavelength 251.619 nm and 212.422 nm. A calibration curve was prepared from a standard solution to convert absorbances into Si concentrations. All analyses were performed in triplicate.

### Statistics analysis of As and Si data

After checking for normality and variance homogeneity in the dataset, three-ways analysis of variance (ANOVA), followed by one-way ANOVAs were applied to As and Si concentrations in fruits, with Confidence Interval (C.I.) of = 95%, and statistical differences between means were deduced using Tukey’s SHD post hoc test, applying a threshold of 0.005. SPSS v23 software (http://www.ibm.com/analytics/us/en/technology/spss/) was used for all analyses.

### Protein extraction, quantification and separation

Proteins were extracted using the phenol buffer-based method described by Faurobert et al. [9]: frozen fruits were ground to powder in liquid nitrogen and a 2 g aliquot suspended in 6 mL 700 mM sucrose, 500 mM Tris-HCl (pH 7.5), 50 mM EDTA, 100 mM KCl, 2% (*w*/*v*) DTT, 0.1% (*v*/v) protease inhibitor cocktail, vortexed and held on ice for 10 min. An equal volume of 500 mM Tris-HCl buffered phenol was then added, the solution mixed for 10 min at room temperature and then centrifuged (5500 *g*, 4 °C, 10 min). The phenolic phase was collected and re-extracted with 3 mL of the same extraction buffer. Proteins were precipitated from the pooled phenolic extract by holding overnight at −20 °C after the addition of five volumes of 0.1 M ammonium acetate (J.T. Baker, Deventer, Holland) saturated in methanol. The proteins were pelleted by centrifugation (5500 *g*, 4 °C, 30 min), and washed first with cold methanol then with cold acetone. Between and after the washing steps, the proteins were re-pelleted by centrifugation (5500 *g*, 4 °C, 30 min), and were finally dried under vacuum. For their quantification, the final protein pellet was dissolved in 200 μL IEF buffer (9 M urea, 4% (*w*/*v*) 3-[(3-cholamidopropyl)dimethylammonio]-1-propanesulfonate), 50 mM DTT, 0.001% (*v*/v) protease inhibitor cocktail, 1% (v/v) pH 3-10 carrier ampholyte mixture) (BioRad), then assayed using a modified Bradford assay [45], taking bovine serum albumin as the standard. The proteins were resolved by two dimensional gel electrophoresis (2DE). For the first dimension 600 μg of the extracted protein was loaded onto a ReadyStrip pH 4-7 11 cm IPG strip (BioRad) which had been rehydrated overnight with 200 μL IEF buffer containing the sample. The IEF step was based on the PROTEAN® i12™ System: the voltage was limited to 250 V for the first 1 h, then raised to 8 KV until 35 KVh had elapsed. The strips were then bathed for 15 min in 3 mL 2% *w*/*v* DTT, 6 M urea, 0.375 M Tris-HCl (pH 8.8), 20% w/v glycerol and 2% w/v SDS, followed by a 15 min incubation in 3 mL 2.5% w/v iodoacetamide, 6 M urea, 0.375 M Tris-HCl (pH 8.8), 2% w/v glycerol. The second dimension separation (SDS-PAGE) used a 12% Criterion XT™ Bis-Tris gel set in a Criterion™ Dodeca™ cell (BioRad); the electrolyte used was 1 M 3-(N-morpholino)propanesulfonic acid, 1 M Tris, 20 mM EDTA, 2% w/v SDS. The gels were stained in QC Colloidal Coomassie G-250 (BioRad). Each assay included three biological replicates, each of which represented three fruits of equivalent ripening stage, harvested from three different plants.

### Spot (feature) analysis

2DE gel images were scanned using the ChemidocMP Imaging System (BioRad) and the images were processed and analyzed using PDQuest v8.0.1 software (BioRad) and checked manually. Feature densities were normalized via a localized regression method and subsequently against the whole gel density. Features were fully analyzed only when detected in all three replicates gels. The density of each feature was averaged over the three replicates and a Student’s *t* test analysis performed applying a threshold of 0.05 to test for significance in abundance between the three treatments (nt, As and As + Si; see Additional file 1: Figure S1A, B). Gel portions containing selected features were excised from the gel using an EXQuest Spot Cutter (BioRad), destained by soaking for 30 min in a 1:1 solution of 100 mM ammonium bicarbonate and acetonitrile (J.T. Baker, Deventer, Holland) and digested with trypsin following the Shevchenko et al. [46] protocol.

### Protein identification

Tryptic peptides were desalted and concentrated by passing through a Zip-Tip C18 (Millipore Corporation, Billerica, MA, USA), according to the manufacturer’s protocol, then dispersed into an α-cyano-4-hydroxycinnamic acid (4-HCCA) matrix, prepared by dissolving 4-HCCA in 50% acetonitrile/0.05% trifluoroacetic acid and spotted on a MALDI plate. The samples were subjected to analysis by a model 4800 MALDI-TOF/TOF™ MS analyzer (Applied Biosystems, Foster City, CA, USA). Peptide mass spectra were acquired in reflectron mode (500-4000 *m/z* range) and analyzed with the help of mMass v5.5 open source software (http://www.mmass.org/). For each feature, a peak list was created and then manually checked for the presence of signal from the matrix complex, trypsin and human keratin peptides. The identification parameters were: trypsin digest: one missed cleavage, mass type: monoisotopic, peptide tolerance: 100 ppm, cysteine carbamidomethylation (fixed) and methionine oxidation (variable modification). Peptide mass fingerprinting analysis was carried out using Mascot v2.3 and 2.4 software (http://www.matrixscience.com/), and proteins identified by means of a search of the non-redundant Viridiplantae protein set within the UniProtKB-Swissprot (version 2015_04x to 2015_12) and Plant EST (version Plants_EST EST_123) (http://www.uniprot.org/) databases. Details regarding protein identification and abundance are given in Table 1, Additional file 2: Table S1 and Additional file 3: Table S2.

**Table 1:** The As and Si content of cv. Aragon and cv. Gladis fruits

			As (μg g^−1^ dw) mean S.E.		Si (μg g^−1^ dw) mean S.E.	
Aragon	NT	t0	0.00^a^	0.00	98.60^a^	4.21
		t14d	0.00 ^a^	0.00	78.95^c^	2.76
	As	t0	0.00 ^a^	0.00	98.60^a^	4.21
		t14d	1.80^b^	0.06	82.35^bc^	0.21
	As + Si	t0	0.00 ^a^	0.00	98.60^a^	4.21
		t14d	1.36^c^	0.04	86.40^bc^	1.56
Gladis	NT	t0	0.00 ^a^	0.00	94.10^a^	2.85
		t14d	0.00 ^a^	0.00	72.95^b^	0.49
	As	t0	0.00 ^a^	0.00	94.10^a^	2.85
		t14d	0.10^b^	0.04	75.85^b^	2.33
	As + Si	t0	0.00 ^a^	0.00	94.10^a^	2.85
		t14d	0.68^c^	0.06	85.55^c^	0.07

Elements concentrations prior to the treatment (t0) and after 14 days of exposure to either the As or the As + Si treatment (t14d). Values shown in the form mean ± S.E. (*n* = 3). Values equal to 0 means BDL. Different superscript letters (a through c) within a column identify means differing significantly from one another (p ≤ 0.05)

### Data mining and analysis

Heat maps of selected proteins were generated by R v3.3.1 (www.r-project.org).

In modern plant biology, the most widely used ontologies are the Gene Ontology (GO) and Gene Ontology MapMan [47].

MapMan Ontology was performed using the GoMapMan tool [47] based on ITAG Release 2.3 (2011-04-26) of the tomato genome sequence (solgenomics.net/organism/Solanum_lycopersicum/genome). MapMan 3.6.0RC1 software (mapman.gabipd.org/web/guest/mapman-download) was used to place proteins within a likely pathway.

## Results

### The As and Si content of tomato fruit

Arsenic concentrations detected in fruits were in accordance to the literature, even allowing for variability among cultivars [48–50]. Also Si concentrations, though seldom measured within tomato fruit, were in keeping with the literature [51, 52].

The As content of cv. Aragon fruits harvested from plants subjected to the As + Si treatment was significantly lower than that of fruits harvested from plants subjected to the As treatment, but the opposite was the case for cv. Gladis fruit (Table 1, Additional file 4: Table S3). Taken across all three treatments, the As content was at least twice as high in cv. Aragon than in cv. Gladis fruits. With respect to the fruits’ Si content, concentrations were consistently higher in cv. Aragon than in cv. Gladis fruits harvested from plants exposed to either the As or the nt treatments, but this cultivar difference disappeared in fruits set by plants exposed to As + Si (Table 1). Si concentrations within the fruits significantly decreased during time for the NT and the As treated plants, but not for the As + Si. In particular, at t14d, Si concentrations were consistently higher in Aragon for NT and As treatments, but not significantly different among the two cvs for the As + Si treatment (Table 1 and Additional file 4: Table S3). Apparently, at t14d, As treatment alone competed with Si for allocation within the fruits, but the concomitant administration of Si with As resulted in an increase of Si, in agreement with its role as a protecting agent against stressors, in this case As [43].

### The tomato fruit proteome

**Fig. 1:**
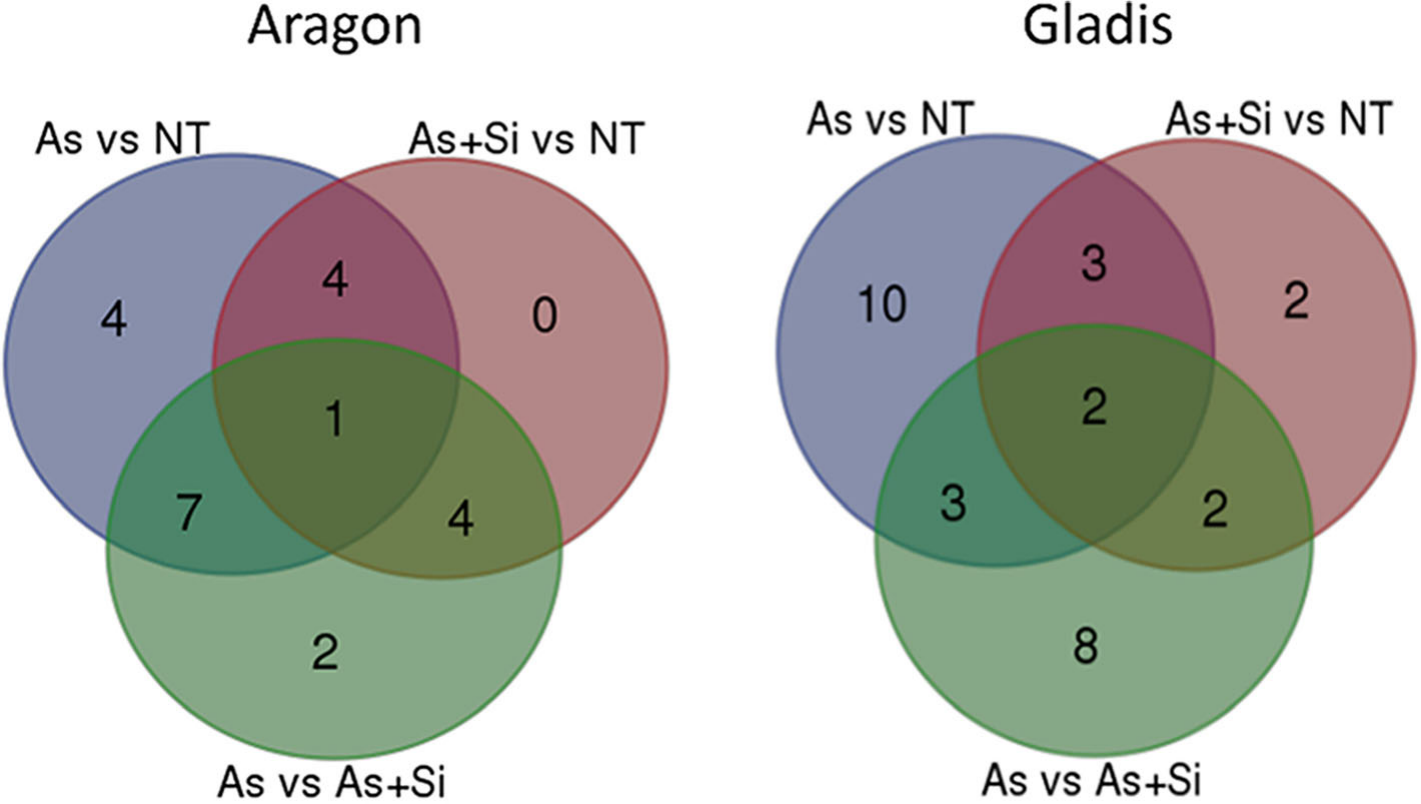
Full set of reprogrammed proteins. The sets of fruit proteins differing significantly (*p* < 0.05) in their abundance in the treatment contrasts nt vs As, nt vs As + Si and As vs As + Si

The 2DE profiling generated overall about 900 visible features for each cultivar. To allow consistent MALDI-TOF analysis the effective number of the reproducible spots in the fruit proteome of cv. Aragon and in that of cv. Gladis were 396 and 349 respectively (Additional file 5: Figure S2A, B). Among the former set, 22 varied in intensity in response to the treatments: 16 of these were identified from the contrast nt vs As, 9 from the contrast nt vs As + Si and 14 from the contrast As vs As + Si; among the cv. Gladis set, the number of variable features was 30, distributed among the three contrasts as, respectively 18, 9 and 15 (Fig. 1). Only 4 of the variable features were common to both cultivars, namely an F-box/WD-40 repeat-containing protein (At5G21040-like), a growth-regulating factor 9-like protein, alcohol dehydrogenase 2 and an ADP/ATP translocator. The identity of the two sets of variable features is given in Table 2, and the associated heat maps illustrating their relative abundance are shown in Additional file 6: Figure S3. Their MapMan ontology BIN assignations (covering 35 BINs) are listed in Additional file 4: Table S3.

**Table 2:** Full set of fruit proteins differentially abundant in (A) cv. Aragon, (B) cv. Gladis

^a^spot n°	^b^Protein name	^c^Gene name	^d^Organism	^e^Score	^f^Cov. %	^g^Exp. Mr. (kDa)/pI	^h^Th. Mr. (kDa)/pI	^i^BLAST protein	^l^ gene name	^m^identity %	^n^Th. Mr. (kDa)	^o^Cov. %
A)												
202	UPF0725 protein At1g27860	At1g27860	*A. thaliana*	31	22	31/4,5	33,4/5,41	uncharacterized protein LOC101252956 (predicted)	Solyc03g005820.2	29	25,5	34
1204	14-3-3 protein 2	Solyc12g057110.2	*S. lycopersicum*	32	22	30/4,5	29,0/4,72					
2103	calmodulin-like protein 3	CML3	*O. sativa*	24	44	23/5,0	20,3/4,62	calmodulin	Solyc03g098050.2	65	16,8	84
3405	actin-51 (Fragment)	Solyc11g005330.1	S. Lycopersicum	110	41	40/5,5	37,3/5,28					
3602	probable lipid-A-disaccharide synthase, mitochondrial	LPXB	A. thaliana	27	7	55/5,5	51,9/9,0	probable lipid-A-disaccharide synthase, mitochondrial (predicted)	Solyc07g049740.2	60	51,8	94
4302	annexin p34	Solyc04g073990.2	S. Lycopersicum	100	88	35/5,5	35,7/6,41					
4803	ATP-dependent Clp protease ATP-binding subunit clpA homolog CD4B, chloroplastic	Solyc12g042060.1	S. Lycopersicum	28	28	>97/5,5	102,5/5,8					
5004	pathogenesis-related protein STH-2-like	Solyc09g090980.2	S. Lycopersicum	78	98	18/5,5	17,2/5,5					
5305	cyclin-D6-1	CYCD6-1	*T. aestivum*	42	12	35/5,8	34,6/5,5	putative cyclin-D6-1	Solyc07g054950.1	33	35,4	80
5602	seed biotin-containing protein SBP65	SBP65	*P. sativum*	75	42	60/5,8	59,6/5,9	seed biotin-containing protein SBP65-like isoform X1	Solyc10g008040.2	34	66,1	62
5702	flavonol sulfotransferase-like	*N/A*	*F. bidentis*	68	49	66/6,0	36,2/5,8	cytosolic sulfotransferase 12-like (predicted)	Solyc05g011870.1	45	38,6	98
5706	nucleolin 2	NUCL2	A. thaliana	41	22	70/6,2	69,0/5,0	nucleolin 1 isoform X1 (predicted)	Solyc02g021220.1	47	65,7	53
6101	eukaryotic translation initiation factor 5A-2	Solyc07g005560.2.1	S. Lycopersicum	89	76	20/6,5	17,7/5,8					
6102	small heat shock protein, chloroplastic	Solyc03g082420.2	S. Lycopersicum	67	56	25/6,3	26,3/7,8					
6103	PHD finger protein ALFIN-LIKE 3	OsI_14081	O. sativa	50	44	25/6,5	28,3/5,7	PHD finger protein ALFIN-LIKE 2-like (predicted)	Solyc10g076690.1	70	27	93
6303	ADP, ATP carrier protein 2, mitochondrial	ANT-G2	T. aestivum	47	48	35/6,2	35,9/9,0	ADP/ATP translocator	Solyc11g062130.1	88	41,9	98
7103	protein LURP-one-related 1	At1g33840	A. thaliana	41	37	23/6,6	25,7/8,6	protein LURP-one-related 10-like (predicted)	Solyc10g085420.1	53	23,8	82
7503	alcohol dehydrogenase 2	ADH2	S. Lycopersicum	49	49	46/6,8	41,8/6,0					
7705	F-box/WD-40 repeat-containing protein At5g21040	At5g21040	A. thaliana	40	33	66/6,7	61,1/8,5	F-box/WD-40 repeat-containing protein At5g21040-like (predicted)	Solyc06g073650.1	59	60,9	100
8105	growth-regulating factor 10	GRF10	O. sativa	43	16	22/6,8	22,5/9,0	growth-regulating factor 9-like (predicted)	Solyc08g079800.2	59	24,6	51
8202	VQ motif-containing protein 4	VQ4	A. thaliana	39	32	28/6,7	26,96/9,8	uncharacterized protein LOC101257285 (predicted)	Solyc02g078030.1	62	25,1	79
8701	V-type proton ATPase catalytic subunit	*N/A*	*B. vulgaris*	40	37	66/6,8	68,8/5,1	vacuolar H + −ATPase A2 subunit isoform	Solyc06g063330.2	92	68,5	100
B)												
302	E3 ubiquitin-protein ligase CHIP	*CHIP*	A. thaliana	27	42	35/4,7	32,1/6,1	E3 ubiquitin-protein ligase CHIP (predicted)	Solyc06g083150.2	69	31,6	97
502	F-box protein At4g00755	*At4g00755*	A. thaliana	24	22	44/4,4	43,1/4,9	F-box protein At4g00755-like (predicted)	Solyc03g026170.2	54	41,1	81
1003	Heavy metal-associated isoprenylated plant protein	*HIPP26*	A. thaliana	26	35	15/5,0	17,2/9,3	heavy metal-associated isoprenylated plant protein 26-like (predicted)	Solyc01g111600.2	85	17,2	100
1305	GDSL esterase/lipase EXL4	*EXL4*	A. thaliana	18	17	40/5,1	38,3/9,0	GDSL esterase/lipase EXL3-like (predicted)	Solyc04g082390.2	42	43,7	91
1401	SKP1-like protein 21	*ASK21*	A. thaliana	18	18	40/5,0	40,3/5,6	SKP1-like protein 21 isoform X1 (predicted)	Solyc06g036070.2	65	40,1	96
1803	heat shock 70 kDa protein	*HSP70*	*Z. mays*	36	26	70/5,2	70,8/5,22	heat shock cognate 70 kDa protein 1	Solyc10g086410.2	89	70,9	99
2802	cell division cycle protein 48 homolog	*CDC48*	*G. max*	69	33	97/5,3	90,5/5,2	cell division cycle protein 48 homolog (predicted)	Solyc06g074980.2	93	90,1	100
2805	sucrose synthase 7	*SUS7*	O. sativa	25	9	97/5,3	98,4/8,0	sucrose synthase 7-like (predicted)	Solyc02g081300.2	69	96,2	98
3302	dihydroflavonol-4-reductase	*Solyc02g085020.2.1*	S. Lycopersicum	24	25	35/5,4	42,7/6,0					
3401	1-aminocyclopropane-1-carboxylate oxidase homolog isoform 1	*Solyc09g089580.2.1*	S. Lycopersicum	71	38	40/5,5	40,9/5,6					
3601	ATP synthase subunit beta, chloroplastic	*Solyc01g007320.2.1*	S. Lycopersicum	48	43	50/5,4	53,5/5,3					
3801	subtilisin-like protease SBT1.7	*SBT1.7*	A. thaliana	26	15	97/5,5	80,1/5,9	SBT1 protein precursor	Solyc04g078110.1	64	78,6	97
4702	calcium-dependent protein kinase 18	*CPK18*	A. thaliana	48	22	64/5,6	60,4/8,7	calcium-dependent protein kinase	Solyc03g033540.2	80	63,7	89
5303	ADP,ATP carrier protein 2, mitochondrial	*ANT-G2*	T. aestivum	47	48	35/6,2	35,9/9,0	ADP/ATP translocator	Solyc11g062130.1	88	41,9	98
5601	enolase	*Solyc09g009020.2.1*	S. Lycopersicum	147	64	48/5,8	48,0/5,7					
6502	alcohol dehydrogenase 2	*Solyc06g059740.2.1*	S. Lycopersicum	31	34	42/6,4	41,7/6,0					
6606	CBL-interacting protein kinase 14	*CIPK14*	O. sativa	37	26	49/6,4	50,5/9,0	CBL-interacting protein kinase	Solyc05g052270.1	60	49,7	100
6607	26S protease regulatory subunit 7 homolog A	*RPT1A*	A. thaliana	45	25	50/6,4	48,2/6,3	26S protease regulatory subunit 7 homolog A-like (predicted)	Solyc06g063140.2	98	47,7	99
6701	phosphomethylpyrimidine synthase, chloroplastic	*THIC*	A. thaliana	69	30	70/6,4	72,6/6,0	phosphomethylpyrimidine synthase, chloroplastic (predicted)	Solyc06g006080.2	83	72,5	99
7203	SufE-like protein 2, chloroplastic	*SUFE2*	A. thaliana	26	23	28/6,5	29,1/9,0	sufE-like protein 2, chloroplastic isoform X2 (predicted)	Solyc05g013710.2	50	29,9	91
7603	cytochrome P450 85A1	*CYP85A1*	A. thaliana	28	34	55/6,5	54,1/9,1	cytochrome P450 85A1	Solyc02g089160.2	70	53,5	96
7605	mannan endo-1,4-beta-mannosidase 3	*Solyc12g013750.1*	S. Lycopersicum	34	24	48/6,5	45,3/5,6					
7701	linoleate 9S-lipoxygenase B	*Solyc01g099190.2.1*	S. Lycopersicum	74	22	60/6,4	97,5/5,6					
7702	argininosuccinate lyase, chloroplastic	*At5g10920*	A. thaliana	28	23	60/6,4	57,9/5,6	argininosuccinate lyase	Solyc04g076320.2	80	nd	89
7703	F-box/WD-40 repeat-containing protein At5g21040	*At5g21040*	A. thaliana	40	33	66/6,7	61,1/8,5	F-box/WD-40 repeat-containing protein At5g21040-like (predicted)	Solyc06g073650.1	59	60,9	100
7704	zinc finger CCCH domain-containing protein 46	*At3g51950*	A. thaliana	29	23	60/6,5	60,2/6,5	zinc finger CCCH domain-containing protein 46-like isoform X2 (predicted)	Solyc01g008600.2	46	61,9	79
7705	pentatricopeptide repeat-containing protein At1g15480, mitochondrial	*At1g15480*	A. thaliana	29	17	66/6,4	67,8/6,4	pentatricopeptide repeat-containing protein At1g80270, mitochondrial (predicted)	Solyc12g020050.1	77	70,1	59
8107	growth-regulating factor 10	*GRF10*	O. sativa	43	16	22/6,8	22,5/9,0	growth-regulating factor 9-like (predicted)	Solyc08g079800.2	59	24,6	51
8205	40S ribosomal protein S3a-2	*RPS3AB*	A. thaliana	29	35	28/6,7	29,9/9,7	40S ribosomal protein S3a (predicted)	Solyc06g072490.2	84	29,6	92
8304	aldo-keto reductase family 4 member C11	*AKR4C11*	A. thaliana	37	18	35/6,6	35,2/6,5	aldo-keto reductase family 4 member C9 (predicted)	Solyc00g015750.1	78	35,0	100

Proteins abundance varied in response tothe As and/or As + Si treatments. Notes: ^a^ spot number assigned following 2DE separation, ^b^ putative protein identification, ^c^ encoding gene of the closest match in the Swiss-Prot database, ^d^ organism, ^e^ score, ^f^ percentage of coverage of the matching peptide sequence tags derived from MASCOT analysis, ^g^ estimated mass and pI, ^h^ predicted mass and pI of the closest match, ^i^ best BLAST hit in tomato, ^l^ gene name, ^m^ predicted mass, ^n^ % identity, ^o^ % coverage identified by BLASTp search of tomato

**Fig. 2:**
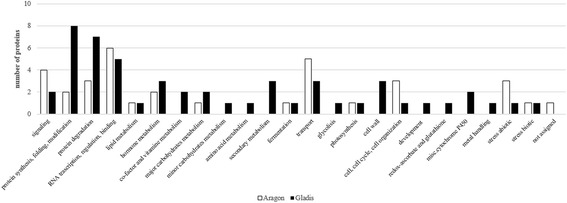
Responsive proteins according to MapMan ontology. The distribution of responsive proteins in the fruit of cv. Aragon and cv. Gladis, according to MapMan ontology classification

**Fig. 3:**
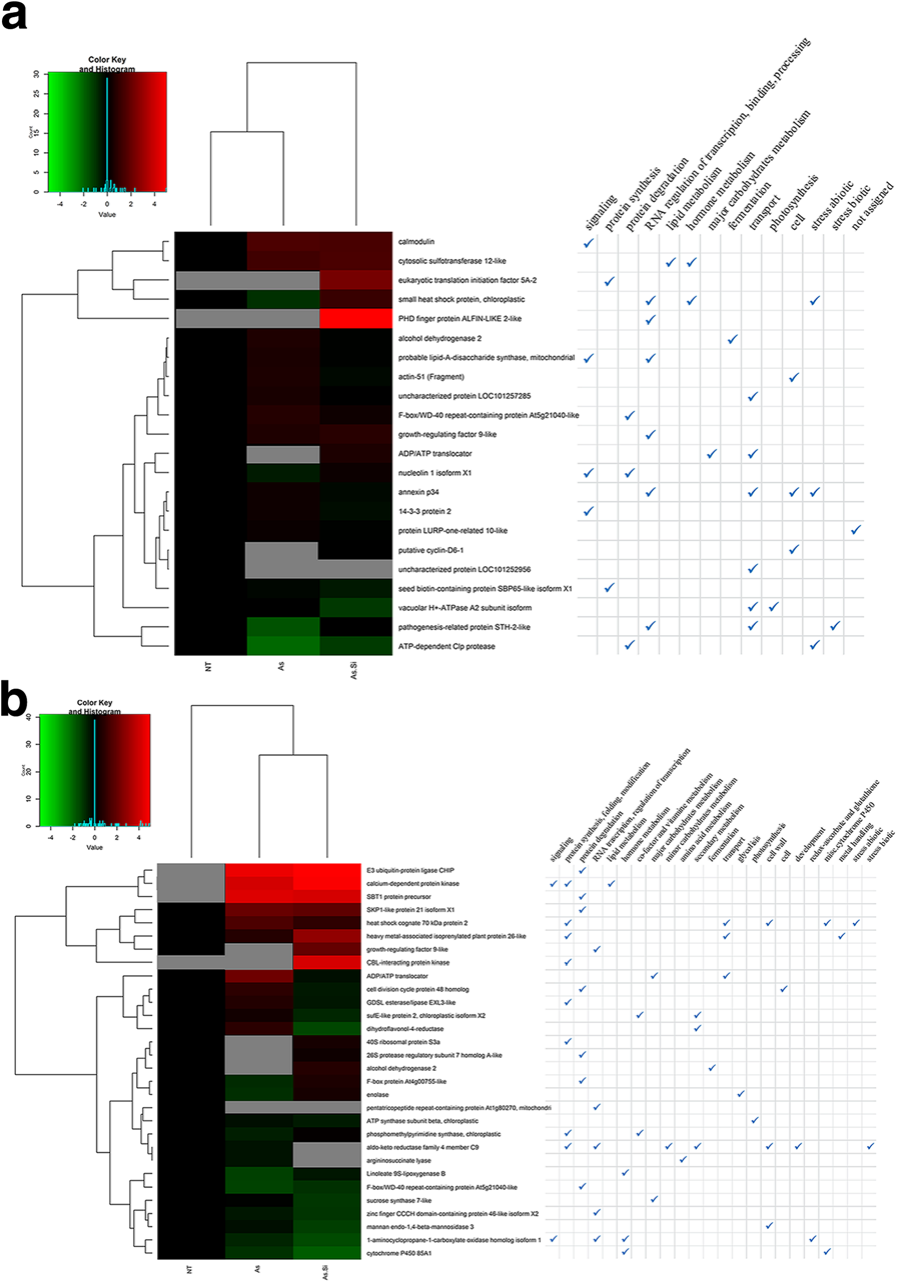
Heat maps of responsive proteins identified in fruits of (**a**) cv. Aragon, (**b**) cv. Gladis. BIN categories of each protein, derived from MapMan ontology, are provided. Proteins showing an enhanced abundance as a result of exposure to either the As and/or As + Si treatment are indicated in *red*, while those showing a reduced abundance are indicated in *green.* Non-responsive proteins are indicated in *black.* Proteins not detected are indicated in *grey*

As shown in Fig. 2a and b, the set of reprogrammed fruit proteins in both cultivars were associated with signaling, protein synthesis, protein degradation, RNA regulation of transcription, hormone metabolism, lipid metabolism, major carbohydrate metabolism, fermentation, photosynthesis, cell, abiotic stress and biotic stress. The BIN RNA binding, RNA processing and transport were found only in cv. Aragon’s fruit proteins; protein folding, protein modification, RNA transcription, co-factor and vitamin metabolism, minor carbohydrate metabolism, amino acid metabolism, secondary metabolism, transport, glycolysis, cell wall, development, redox-ascorbate and glutathione, miscellaneous cytochrome P450 and metal handling were found only in cv. Gladis’ fruit proteins. Their abundance and function are shown in Fig. 3a and b. Further details are provided in Additional file 4: Table S5A and B.

## Discussion

### Fruit proteins reprogrammed by As and/or As + Si treatments in cv. Aragon

The stress-induced reprogramming of proteins involved in transcription, signaling and hormone metabolism, transport and cell may have arisen as a result of the impact of the treatments on the ripening process, which involves extensive changes to the transcriptome, proteome and metabolome [2, 3, 9] (Figs. 2 and 3a, Additional file 4: Table S5A). The As treatment affected transcription-, signaling- and hormone-related proteins, all of which are involved in the direct and retrograde communication between the nucleus and the organelles; their modulation could well disturb the normal development of the fruit [10, 53]. Proteins related to transport and cell metabolism, also essential for ripening, probably served as a means for the tissue to sequester As into non-essential cellular structures and to increase the concentration of antioxidants needed to maintain an appropriate cellular redox state [54]. Supplementation with Si had a marked influence over the abundance of proteins associated with transcription, signaling and hormone metabolism, as also noted in the interaction of tomato with its pathogen *Ralstonia solanacearum* [55]. The protective action of Si against pathogen attack has been characterized by an accumulation of various defense-associated transcription factors (here, members of the WRKY family), signaling proteins (calmodulin and 14-3-3 protein 2) and the modulation of endogenous hormone synthesis [56]. An altered abundance of proteins determining cell shape and the cytoskeleton (such as actin and annexin) has been shown to be associated with ripening [8–11]. Some of these were induced by the As treatment, presumably because of the oxidative stress thereby imposed, which in turn was likely to have encouraged the peroxidation of membrane lipids, leading to intra-cellular electron leakage [57]. Si has a suppressive effect on cellular free radical content, thereby alleviating lipid peroxidation [58]. A reduced need for cell shape adjustment following the Si-enabled stabilization of cell membranes and reduction in free radical content could explain the fall in abundance of proteins related to cell shape and structure development. A possible detrimental effect of As on the ripening process may have been exerted by the lowered abundance of Clp protease CD4B and the small heat-shock protein HSP21, both of which are essential for, respectively, chromoplast development and fruit color change [11]; the abundance of both these proteins was enhanced by the provision of Si.

### Fruit proteins reprogrammed by As and/or As + Si treatments in cv. Gladis

The abundance of several proteins belonging to the ubiquitin-proteasome system (UPS), an essential component of the regulatory network controlling the abundance of a number of enzymes, along with certain structural and regulatory proteins [59], was affected by the treatments (Figs. 2 and 3b, Additional file 4: Table S5B). The added proteomic flexibility provided by the UPS provides a means of adapting to an episode of abiotic stress [60]. The abundance of the three hormone synthesis-associated proteins 1-aminocyclopropane-1-carboxylate oxidase, cytochrome P450 85A1 and linoleate 9S-lipoxygenase B was lower in fruits harvested from plants subjected to both the As and the As + Si treatments than in plant from the nt treatment. The 26S proteasome complex is of particular interest in the context of the plant response to As, because in *A. thaliana*, the F subunit of the 20S core particle has been shown to be responsible for the increased As tolerance shown by the *ars5* mutant [61]. Here, the reprogramming concerned a subunit of the 19S rather than the 20S particle. Wang et al. [53] have shown that 12 of the 13 proteins represented in the 26S proteasome complex are highly abundant in ripe tomato fruits. Supplementation with Si was unable to restore the level of these proteins to the nt level, perhaps because the fruits’ As concentration was higher in the As + Si than in the As treatment, resulting in a higher As/Si ratio (Table 1). Proteins related to protein synthesis, folding and modification were affected to a different degree by the two treatments: the pattern was inconsistent, probably because the abundance of these proteins is so strongly influenced by the ripening process [53, 62]. In particular, AKR (aldo/keto reductase), an enzyme known to contribute to abiotic stress tolerance [63], typically increases during the course of early fruit development, and declines during the red ripening stage [64]. AKR was under-abundant in the As treatment and undetectable in the As + Si treatment, indicating a possible acceleration in the ripening process induced by both treatments. AKR contributes to the detoxification of malondialdehyde [65], so its suppression in the As + Si treatment is consistent with the protective action of Si against lipid peroxidation. Several proteins involved in carbon metabolism and ATP production were under-abundant in the As treatment, in accordance with the documented detrimental effect of this element on respiration and photosynthesis [17, 23]. The concentration of the glycolysis enzyme enolase was substantially raised by Si supplementation, in keeping with the proposed contribution of Si to the alleviation of As stress [43]. The abundance of a number of secondary metabolism-related proteins was noted in the fruits of the As treated plants, an effect which was reversed in the As + Si treatment. A sharp increase in secondary metabolite content is a feature of fruit maturation, as the plant needs to deal with a rise in oxidant activity [9, 11]; in this regard both treatments appeared to perturb the amount of enzymes required during the fruit ripening process. Proteins related to cell wall structure are of relevance because both As and Si can affect cell wall organization [66, 67]. Si contributes to the alleviation of stress, not just via its enhancement of cell wall deposition [68], but also by its stimulation of cell wall component synthesis [69]. Structural changes to the cell wall during ripening involve pectins, hemicelluloses and cellulose [70]. In the tomato fruit, the expression level of a gene encoding endo-β-mannanase has been shown to increase markedly at the breaker stage and to remain high throughout the red and over-ripe stages [71]. This enzyme has also been found to accumulate in response to parasite attack, suggesting its active involvement in the stress response [72]. It is thus possible that Si influences the concentrations of this and similar proteins within its broader priming effect mentioned above. Here, the under-abundance of mannosidases may have induced an increase in the cellulose content, tightening cellulose networks and strengthening cell wall stability.

### MapMan pathways identified in tomato fruit under As and As + Si treatment

**Fig. 4:**
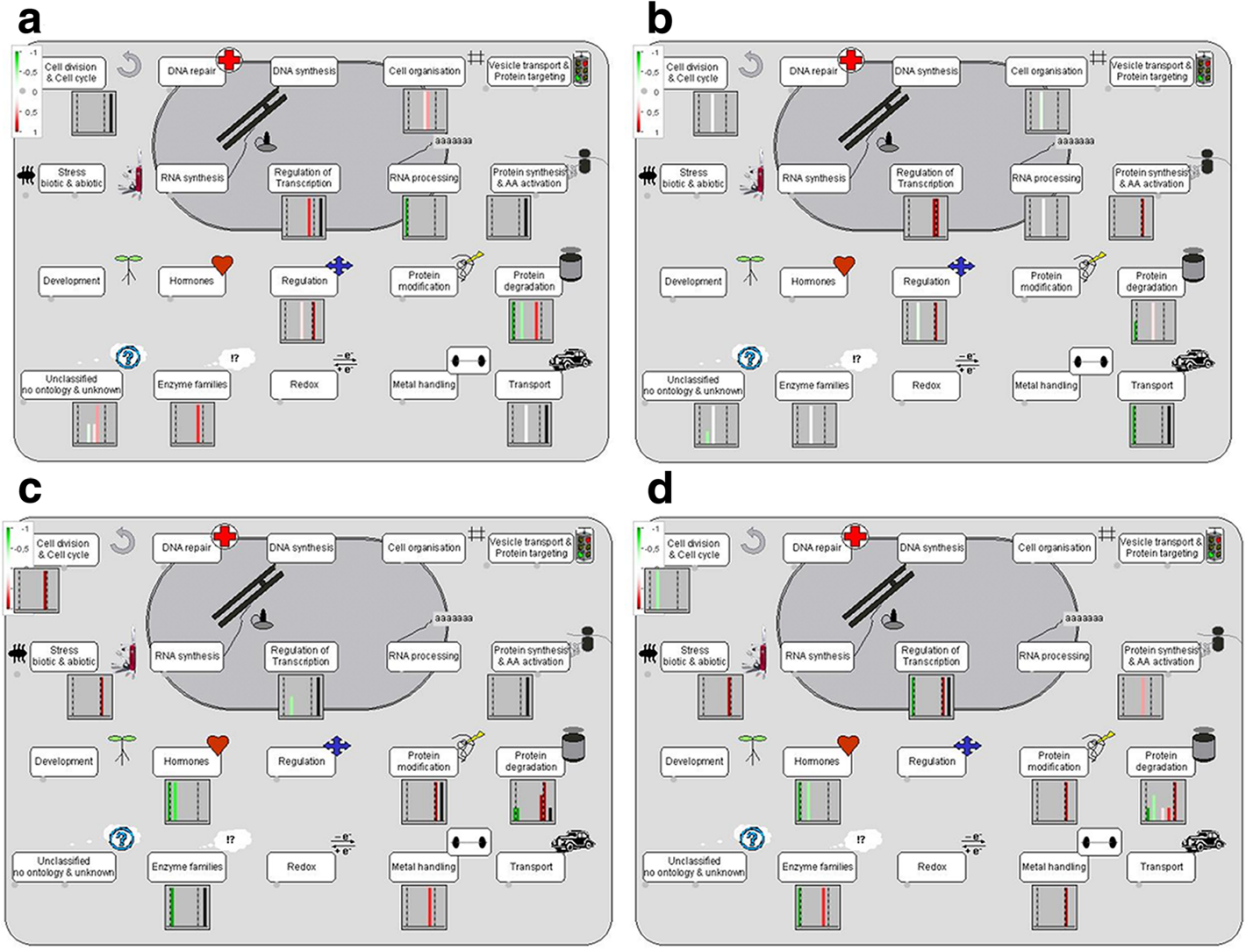
MapMan Cell function overview of the differentially abundant tomato fruit proteins. Cell function overview of the differentially abundant tomato fruit proteins in (**a**, **b**) cv. Aragon, (**c**, **d**) cv. Gladis in response to (**a**, **c**) the As and (**b**, **d**) the As + Si treatmentsProteins showing an enhanced or a reduced abundance as a result of the treatment are marked in, respectively, *red* and *green*. Proteins not detected are indicated in *black*

Metabolic pathways associated with the cellular status of the fruits set by cvs Aragon and Gladis were derived using MapMan software, based on the Slyc_ITAG2.3 database (solgenomics.net/gb2/gbrowse/ITAG2.3_genomic/). The highest scoring processes (Fig. 4, Additional file 7: Figure S4, Additional file 8: Figure S5) were “cell function overview” and “biotic stress pathway” for both cultivars, with the addition of “proteosome” for cv. Gladis.

The “cell function overview” assigned 19 of the 21 mapped proteins in the cv. Aragon fruits to nine processes, namely: “cell division and cell cycle”, “regulation of transcription”, “protein synthesis and amino acid activation”, “protein degradation”, “enzyme families”, “cell organization”, “RNA processing”,, “regulation”, and “transport” (Fig. 4a, b). Of the 30 proteins in cv. Gladis 21were assigned within the “cell function overview” to the first five categories mentioned for cv. Aragon. The reminder proteins were assigned to: “stress biotic and abiotic”, “hormones”, “protein modification”, and “metal handling” (Fig. 4c, d). The 14 proteins assigned to the “biotic stress pathway” were associated with “hormone signaling”, “cell wall”, “proteolysis”, “heat shock proteins” and “secondary metabolites”, while of the eight assigned to “proteolysis”, six were associated with the “proteasome” (Additional file 8: Figure S5). A subset of the proteins identified from fruits harvested from plants of the two cultivars subjected to either the As or the As + Si treatment belonged to the same cell function processes, namely “cell division and cell cycle”, “regulation of transcription”, “protein synthesis and amino acid activation”, “protein degradation” and “enzyme families”. Overall, those reprogrammed in cv. Aragon were largely associated with the regulation of transcription, cell organization, transport and RNA processing, while those in cv. Gladis were involved in protein synthesis, transformation, ubiquitination and proteolysis and hormones. The MapMan pathway analysis highlighted the distinct strategies adopted by the two cultivars to cope with As-induced stress, as well as the different ways in which the provision of Si mitigated these effects. Proteins implicated in the biotic stress response were reprogrammed in the fruits of both cultivars, but there were differences with respect to the major classes of proteins involved (Additional file 7: Figure S4). For example, transcription regulation, cell organization-, signaling- and transport-associated proteins were prominent in cv. Aragon fruits, whereas in cv. Gladis fruits, the main categories were protein modification, protein degradation and hormones (Fig. 4, Additional file 8: Figure S5). The fruits set by cv. Gladis plants subjected to the As or the As + Si treatments featured a higher number of reprogrammed proteins related specifically to the biotic stress response.

### Reprogrammed proteins associated with the stress response

**Fig. 5:**
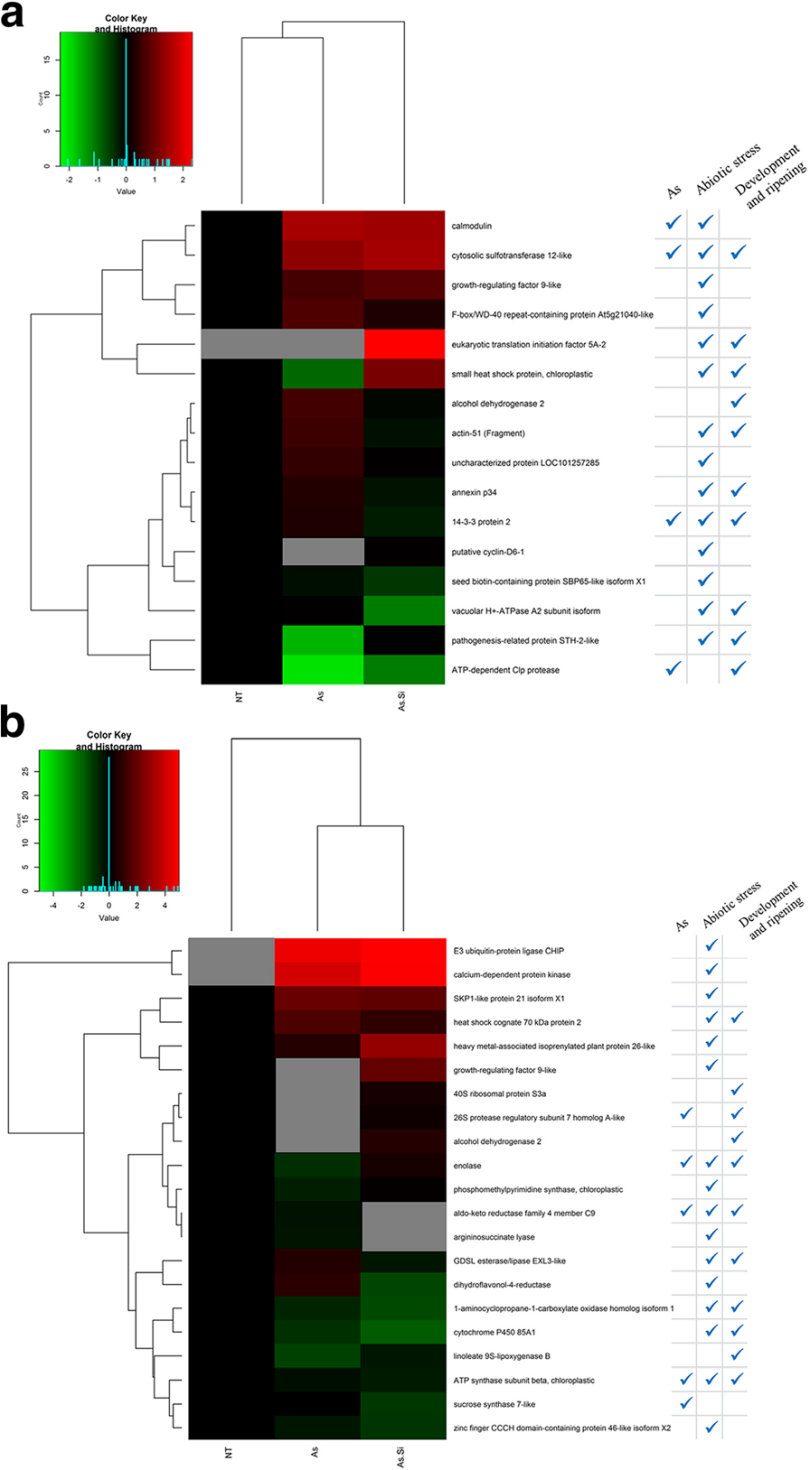
Alternative classification for the differentially abundant fruit proteins in (**a**) cv. Aragon, (**b**). Heat map of the set of differentially abundant fruit proteins identified in (**a**) cv. Aragon, (**b**) cv. Gladis, with an indication of their documented involvement in the As stress response, the abiotic stress response, fruit development or ripening. Proteins showing an enhanced abundance as a result of exposure to either the As and/or As + Si treatment are indicated in *red*, while those showing a reduced abundance are indicated in *green.* Non-responsive proteins are indicated in *black.* Proteins not detected are indicated in *grey*

The two sets of reprogrammed proteins were grouped into sub-sets related to the As stress response, the abiotic stress response, fruit development and ripening (Additional file 4: Table S4); the respective heat maps are given in Fig. 5. In cv. Aragon fruits, four proteins implicated in the As stress response were consistently reprogrammed in response to both the As and the As + Si treatments. Several proteins involved in the abiotic stress response were also affected by ripening, although there are no precedents in the literature for this connection. Four proteins classed as highly abundant during fruit development and ripening [9, 53, 62] were reduced in abundance or suppressed altogether in the fruits harvested from plants subjected to the As treatment, and the addition of Si only partially reversed these effects. The behavior of five cv. Gladis proteins previously implicated in the As stress response [73, 74] was confirmed here, but this was not the case for either ADK or enolase, which have been claimed to be inducible by As exposure by, respectively, Paulose et al. [75] and Tripathi et al. [34], whereas in the present experiment, both were reduced in abundance by the treatment. Five further proteins, which responded to fruit development and ripening according to Faurobert et al. (2007) [9] and Suzuki et al. [8], were here either partially or completely suppressed by the As treatment; however, Si supplementation counteracted the effect for three of the five proteins. Finally, several proteins associated with the abiotic stress response, specifically chloroplastic ATP-dependent Clp protease ATP-binding subunit clpA homolog CD4B and linoleate 9S-lipoxygenase B [76, 77] were modulated by both the As and As + Si treatments, while the abundance of both cytosolic sulfotransferase (involved in the As response, see Komatsu et al., [78]) and sucrose synthase 7-like (general abiotic stress response, see Hirschmann et al., [79]) was here correlated with fruit ripening (Fig. 5, Additional file 4: Table S5).

## Conclusions

The deleterious effect of As and the alleviating role of Si are an interesting example of the complexity in plant-environment interaction. All of these depends on uptake from the media of As and Si, on their transport to the different parts of the plant and on the activation of response mechanisms which are tissue/organ specific. Whereas the effect of As treatment, and its modulation by the supply of Si, on the root, leaf and seed of several plant species has been examined in some detail [23, 33, 80], the present report constitutes a first attempt to investigate the consequences of their presence for the fruit proteome which is highly dynamic because of the many and rapid changes which take place during the ripening process [9, 81]. Despite their apparent genetic similarity, the two tomato cultivars compared here have been shown previously to differ in their response to As and As + Si treatment, for example to the extent to which they accumulate these elements in their fruit [34]. To date, in the tomato genome no orthologous sequence of AtNIP5;1, OsLsi1, OsLsi2 or other As/Si specific transporters has been cloned [82]. Still, the existing competition between As and Si in allocation within the fruit might argue favorably for the existence of common transport mechanisms. We found that only five proteins were related to signal transduction in each cultivar for all treatments (Fig. 3); in general the main class of proteins readjusted by As and As + Si were related to RNA regulation and transcription, transport, and protein degradation, but the two cultivars showed notable differences. In cv. Aragon reprogramming of transcription factors and ATP/ADP transporters indicated that As and As + Si could act on DNA and ribosomal activity. In cv. Gladis, As and As + Si modulated mostly proteins involved in proteins synthesis, folding and degradation, specifically the proteasome complex, bearing out possible alteration in the enzymatic pool acting in the ripening process.

This protein reprogramming in the fruit proteome is accompanied with the biosynthesis of antioxidant and stress protecting metabolites as carotenoids, phenolics, glutathione and ascorbic acid (Marmiroli et al. unpublished data), which contribute to maintain the fruit homeostasis.

## Additional files

Additional file 1: Figure S1. Scatter plot of detected spots on 2D gel: a) Aragon, and b) Gladis. (PDF 536 kb)

Additional file 2: Table S1. MALDI-TOF/TOF data associated with differentially abundant fruit proteins identified in (A) cv. Aragon and (B) cv. Gladis. See attached excels file. (XLSX 58 kb)

Additional file 3: Table S2. Protein abundance data. See attached excels file. (XLSX 28 kb)

Additional file 4: Table S3. Three-ways ANOVA for As and Si concentrations in fruits of tomato cultivars Aragon and Gladis. **Table S4**. MapMan BIN assignation of the differentially abundant fruit proteins in (A) cv. Aragon, (B) cv. Gladis. **Table S5**. Description of the differentially abundant fruit proteins in (A) cv. Aragon, (B) cv. Gladis. (DOCX 90 kb)

Additional file 5: Figure S2. 2D gel electrophoresis images of fruit proteins from A) Aragon, and B) Gladis. (PDF 973 kb)

Additional file 6: Figure S3. Heat map of the differentially abundant proteins present in A) Aragon, B) Gladis. (PDF 737 kb)

Additional file 7: Figure S4. Representation of the differentially Differentially abundant fruit proteins involved in the “Biotic Stress” response MapMan pathway. (PDF 578 kb)

Additional file 8: Figure S5. Representation of the dDifferentially abundant fruit proteins in Gladis involved in the “Ubiquitin Dependent Degradation” MapMan. (PDF 444 kb)

## Abbreviations

2DE: Two dimensional gel electrophoresis
4-HCCA: α-cyano-4-hydroxycinnamic acid
CI: Confidence interval
DTT: Dithiothreitol
EC: Electrical conductivity
EDTA: Ethylenediamine tetra acetic acid
IEF: Isoelectofocusing
ROS: reactive oxygen species
SDS PAGE: Sodium dodecyl sulphate polyacrylamide gel electrophoresis
SDS: Sodium dodecyl sulfate
Treatments: “nt” = non-treated control, “As” = treatment with As only, “As + Si” = treatment with As in combination with Si
