# Supplementary material for: Target proteins reprogrammed by As and As + Si treatments in Solanum lycopersicum L. fruit
Source: BMC Plant Biol. 2017 Nov 21;17:210. doi: 10.1186/s12870-017-1168-2 (PMC5696772; doi:10.1186/s12870-017-1168-2)
Supplement: Supplementary file 1 — Scatter plot of detected spots on 2D gel: a) Aragon, and b) Gladis. (PDF 536 kb) [file 12870_2017_1168_MOESM1_ESM.pdf]

Figure S1: Scatter plot of detected spots on 2D gel: a) Aragon, and b) Gladis

Scatter plot of the spots captured in 2D gel corresponding to differentially abundant fruit proteins when comparing treatments in pairs.

X axis value = intensity % in condition i

Y axis value = intensity % in condition j

Pink band: comprises points whose differential abundance in conditions i and j is less than the fixed threshold ($p<0.05$).

Points outside the gray bands represent the proteins chosen for further MS fingerprinting.

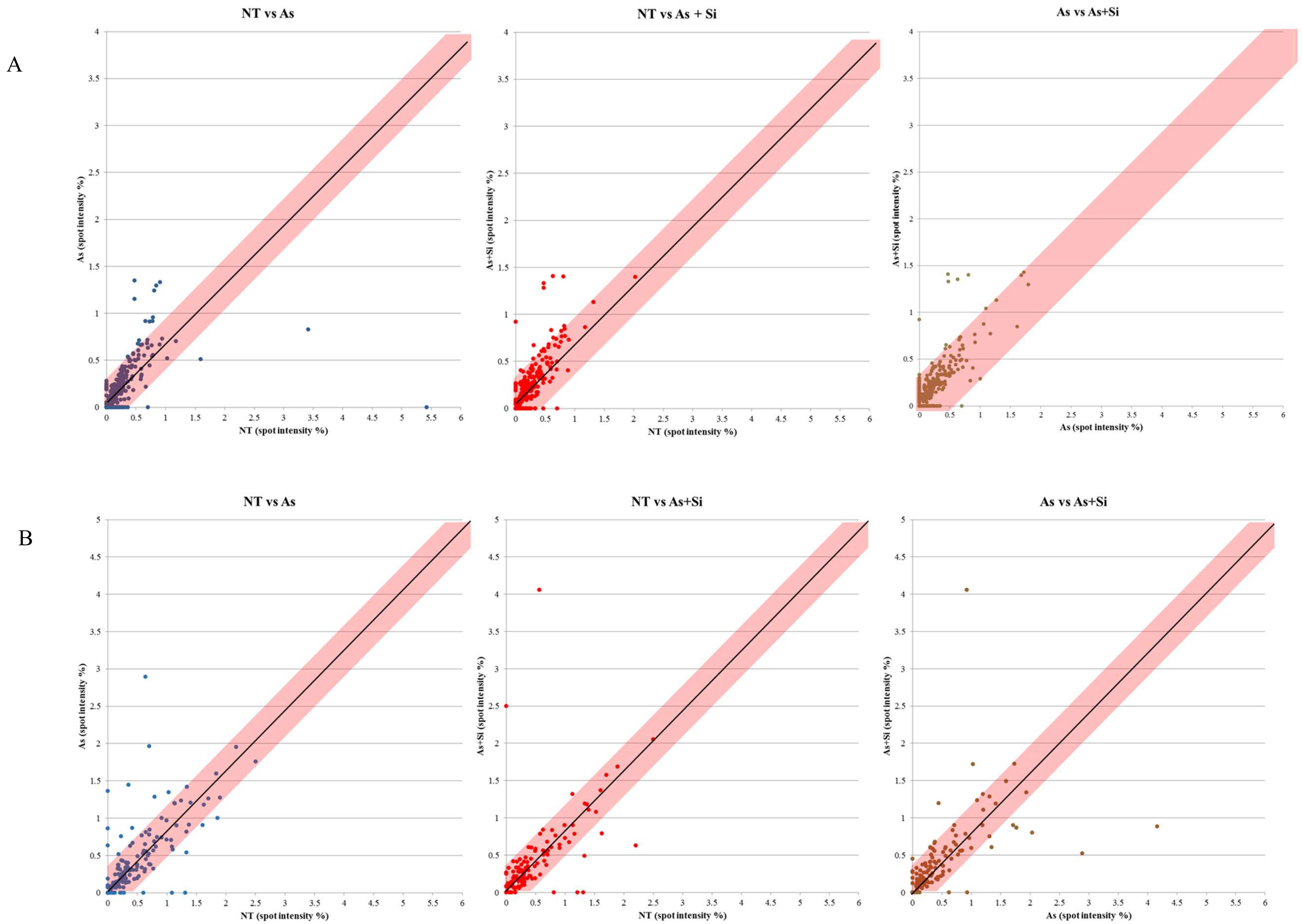
A
NT vs As
As (spot intensity %)
NT (spot intensity %)
NT vs As + Si
As+Si (spot intensity %)
NT (spot intensity %)
As vs As+Si
As+Si (spot intensity %)
As (spot intensity %)
B
NT vs As
As (spot intensity %)
NT (spot intensity %)
NT vs As+Si
As+Si (spot intensity %)
NT (spot intensity %)
As vs As+Si
As+Si (spot intensity %)
As (spot intensity %)
