# Supplementary material for: Target proteins reprogrammed by As and As + Si treatments in Solanum lycopersicum L. fruit
Source: BMC Plant Biol. 2017 Nov 21;17:210. doi: 10.1186/s12870-017-1168-2 (PMC5696772; doi:10.1186/s12870-017-1168-2)
Supplement: Supplementary file 4 — Three-ways ANOVA for As and Si concentrations in fruits of tomato cultivars Aragon and Gladis. Table S4. MapMan BIN assignation of the differentially abundant fruit proteins in (A) cv. Aragon, (B) cv. Gladis. Table S5. Description of the differentially abundant fruit proteins in (A) cv. Aragon, (B) cv. Gladis. (DOCX 90 kb) [file 12870_2017_1168_MOESM4_ESM.docx]

**Additional file 3**

**Table S3: Three-ways ANOVA for As and Si concentrations in fruits of tomato cultivars Aragon and Gladis.**

| **Independent Variables (IVs) Main Effect and Interactions** | **Dependent variables**  **(DVs)** | **Signif.** |
| --- | --- | --- |
| **Cultivar** | As (µg g^-1^) | *** |
|  | Si (mg Kg^-1^) | ** |
| **Treatment** | As (µg g^-1^) | *** |
|  | Si (mg Kg^-1^) | *** |
| **Time** | As (µg g^-1^) | *** |
|  | Si (mg Kg^-1^) | ** |
| **Cultivar * Treatment** | As (µg g^-1^) | *** |
|  | Si (mg Kg^-1^) | ** |
| **Cultivar * Time** | As (µg g^-1^) | *** |
|  | Si (mg Kg^-1^) | * |
| **Treatment * Time** | As (µg g^-1^) | *** |
|  | Si (mg Kg^-1^) | *** |
| **Cultivar * Treatment * Time** | As (µg g^-1^) | *** |
|  | Si (mg Kg^-1^) | *** |

Signif.= significance (*: p≤0.05; **: p≤0.01; ***: p≤0.001; ns= not significant); IVs: Cultivar = Aragon, Gladis; Treatment = NT, As, As+Si; Time = t0, t14d.

**Table S4.** **MapMan BIN assignation of differentially abundant fruit proteins in (A) cv. Aragon, (B) cv. Gladis.**.

A)

| Protein name | Gene name | Bin Code | Bin Name |
| --- | --- | --- | --- |
| 14-3-3 protein 2 | Solyc12g057110.2 | 30.7 | signalling.14-3-3 proteins |
| actin-51 (Fragment) | Solyc11g005330.1 | 31.11 | cell.organisation |
| ADP/ATP translocator | Solyc11g062130.1 | 2.1.2.5, 34.8, 34.9, 34.14 | major CHO metabolism.synthesis.starch.transporter; transport.metabolite transporters at the envelope membrane; transport.metabolite transporters at the mitochondrial membrane; transport.unspecified cations |
| alcohol dehydrogenase 2 | Solyc06g059740.2 | 5.3, 26.11.1 | fermentation.ADH; misc.alcohol dehydrogenases.cinnamyl alcohol dehydrogenase |
| annexin p34 | Solyc04g073990.2 | 20.2.1, 26.7, 27.3.39, 31.1, 34.3 | stress.abiotic.heat; misc.oxidases - copper, flavone etc; RNA.regulation of transcription.AtSR transcription factor family; cell.organisation; transport.amino acids |
| ATP-dependent Clp protease ATP-binding subunit clpA homolog CD4B, chloroplastic | Solyc12g042060.1 | 20.2.1, 29.5.5 | stress.abiotic.heat; protein.degradation.serine protease |
| calmodulin | Solyc03g098050.2 | 30.3 | signalling.calcium |
| cytosolic sulfotransferase 12-like (predicted) | Solyc05g011870.1 | 11.8, 17.3.1.1.5 | lipid metabolism.exotics (steroids, squalene etc); hormone metabolism.brassinosteroid.synthesis-degradation.BRs.metabolic regulation |
| eukaryotic translation initiation factor 5A-2 | Solyc07g005560.2 | 26.8, 29.2.3 | misc.nitrilases, nitrile lyases, berberine bridge enzymes, reticuline oxidases, troponine reductases; protein.synthesis.initiation |
| F-box/WD-40 repeat-containing protein At5g21040-like (predicted) | Solyc06g073650.1 | 26.2, 29.5.11.4.3.2 | misc.UDP glucosyl and glucoronyl transferases; protein.degradation.ubiquitin.E3.SCF.FBOX |
| growth-regulating factor 9-like (predicted) | Solyc08g079800.2 | 27.3.50, 27.3.83 | RNA.regulation of transcription.general transcription; RNA.regulation of transcription.GRF zinc finger family |
| nucleolin 1 isoform X1 (predicted) | Solyc02g021220.1 | 29.5.11.4.2, 30.3 | protein degradation.ubiquitin.E3.RING; signalling.calcium |
| pathogenesis-related protein STH-2-like | Solyc09g090980.2 | 20.1.7, 27.1.19, 34.16 | stress.biotic.PR-proteins; RNA.processing.ribonucleases; transport.ABC transporters and multidrug resistance systems |
| PHD finger protein ALFIN-LIKE 2-like (predicted) | Solyc10g076690.1 | 27.3.2 | RNA.regulation of transcription.alfin-like |
| probable lipid-A-disaccharide synthase, mitochondrial (predicted) | Solyc07g049740.2 | 27.3.44, 30.2.16 | RNA.regulation of transcription.chromatin remodeling factors; signalling.receptor kinases.Catharanthus roseus-like RLK1 |
| protein LURP-one-related 10-like (predicted) | Solyc10g085420.1 | 35.2 | not assigned.unknown |
| putative cyclin-D6-1 | Solyc07g054950.1 | 31.3 | cell.cycle |
| seed biotin-containing protein SBP65-like isoform X1 | Solyc10g008040.2 | 26.8, 29.2.2, 33.2 | misc.nitrilases, nitrile lyases, berberine bridge enzymes, reticuline oxidases, troponine reductases; protein.synthesis.ribosome biogenesis; development.late embryogenesis abundant |
| small heat shock protein, chloroplastic | Solyc03g082420.2 | 17.3.1.1.1, 20.2.1, 27.4 | Hormone metabolism.brassinosteroid.synthesis-degradation.BRs.DET2; stress.abiotic.heat; RNA.RNA binding |
| uncharacterized protein LOC101252956 (predicted) | Solyc03g005820.2 | 34.10 | transport.nucleotides |
| uncharacterized protein LOC101257285 | Solyc02g078030.1 | 34.13 | transport.peptides and oligopeptides |
| vacuolar H+-ATPase A2 subunit isoform | Solyc06g063330.2 | 1.1.4, 34.1.1 | PS.lightreaction.ATP synthase; transport.p- and v-ATPases.H+-transporting two-sector ATPase |

B)

| Protein name | Gene name | Bin Code | Bin Name |
| --- | --- | --- | --- |
| 1-aminocyclopropane-1-carboxylate oxidase homolog isoform 1 | Solyc09g089580.2 | 12.2.99, 16.5.1.1.1.13, 17.5.1.2, 21.2, 23.2, 26.14, 27.2, 30.6, 34.13 | N-metabolism.ammonia metabolism.unspecified; secondary metabolism.sulfur-containing.glucosinolates.synthesis.aliphatic.2-oxoglutarate-dependent dioxygenase; hormone metabolism.ethylene.synthesis-degradation.1-aminocyclopropane-1-carboxylate oxidase; redox.ascorbate and glutathione; nucleotide metabolism.degradation; misc.oxygenases; RNA.transcription; signalling.MAP kinases; transport.peptides and oligopeptides |
| 26S protease regulatory subunit 7 homolog A-like (predicted) | Solyc06g063140.2 | 29.5.11.20 | protein.degradation.ubiquitin.proteasom |
| 40S ribosomal protein S3a (predicted) | Solyc06g072490.2 | 29.2.1.2.1.53, 29.2.2 | protein.synthesis.ribosomal protein.eukaryotic.40S subunit.S3A; protein.synthesis.ribosome biogenesis |
| ADP/ATP translocator | Solyc11g062130.1 | 2.1.2.5, 34.8, 34.9, 34.14 | major CHO metabolism.synthesis.starch.transporter; transport.metabolite transporters at the envelope membrane; transport.metabolite transporters at the mitochondrial membrane; transport.unspecified cations |
| alcohol dehydrogenase 2 | Solyc06g059740.2 | 5.3, 26.11.1 | fermentation.ADH; misc.alcohol dehydrogenases.cinnamyl alcohol dehydrogenase |
| aldo-keto reductase family 4 member C9 (predicted) | Solyc00g015750.1 | 3.5, 10.3, 16.8.2, 20.1.2, 27.3.6, 29.4.1.55, 33.99 | minor CHO metabolism.others; cell wall.hemicellulose synthesis; secondary metabolism.flavonoids.chalcones; stress.biotic.receptors; RNA.regulation of transcription.basic helix-loop-helix family (bHLH); protein.postranslational modification.kinase.receptor like cytoplasmatic kinase V; development.unspecified |
| argininosuccinate lyase | Solyc04g076320.2 | 13.1.2.3.23 | amino acid metabolism.synthesis.glutamate family.arginine.argininosuccinate lyase |
| ATP synthase subunit beta, chloroplastic | Solyc01g007320.2 | 1.1.4.2, 1.1.4.3, 9.9 | PS.lightreaction.ATP synthase.beta subunit; PS.lightreaction.ATP synthase.epsilon chain; mitochondrial electron transport / ATP synthesis.F1-ATPase |
| calcium-dependent protein kinase | Solyc03g033540.2 | 11.9.3.5, 29.4.1, 30.3, 30.4 | lipid metabolism.lipid degradation.lysophospholipases.phosphoinositide phospholipase C; protein.postranslational modification.kinase; signalling.calcium; signalling.phosphinositides |
| CBL-interacting protein kinase | Solyc05g052270.1 | 29.4 | protein.postranslational modification |
| cell division cycle protein 48 homolog (predicted) | Solyc06g074980.2 | 29.5.9, 29.5.11.20, 31.2 | protein.degradation.AAA type; protein.degradation.ubiquitin.proteasom; cell.division |
| cytochrome P450 85A1 | Solyc02g089160.2 | 17.3.1.1.4, 26.10 | hormone metabolism.brassinosteroid.synthesis-degradation.BRs.BR6OX; misc.cytochrome P450 |
| dihydroflavonol-4-reductase | Solyc02g085020.2 | 16.8.3.1 | secondary metabolism.flavonoids.dihydroflavonols.dihydroflavonol 4-reductase |
| E3 ubiquitin-protein ligase CHIP (predicted) | Solyc06g083150.2 | 29.5.11.4.2 | protein.degradation.ubiquitin.E3.RING |
| enolase | Solyc09g009020.2 | 4.1.13 | glycolysis.cytosolic branch.enolase |
| F-box protein At4g00755-like (predicted) | Solyc03g026170.2 | 29.5.11.4.3.2 | protein.degradation.ubiquitin.E3.SCF.FBOX |
| F-box/WD-40 repeat-containing protein At5g21040-like | Solyc06g073650.1 | 26.2, 29.5.11.4.3.2 | misc.UDP glucosyl and glucoronyl transferases; protein.degradation.ubiquitin.E3.SCF.FBOX |
| GDSL esterase/lipase EXL3-like (predicted) | Solyc04g082390.2 | 29.7 | protein.glycosylation |
| growth-regulating factor 9-like | Solyc08g079800.2 | 27.3.50, 27.3.83 | RNA.regulation of transcription.general transcription; RNA.regulation of transcription.GRF zinc finger family |
| heat shock cognate 70 kDa protein 2 | Solyc10g086410.2 | 10.3, 20.2.1, 26.10, 29.6, 34.99 | cell wall.hemicellulose synthesis; stress.abiotic.heat; misc.cytochrome P450; protein.folding; transport.misc |
| heavy metal-associated isoprenylated plant protein 26-like | Solyc01g111600.2 | 15.2, 29.4, 34.99 | metal handling.binding, chelation and storage; protein.postranslational modification; transport.misc |
| linoleate 9S-lipoxygenase B | Solyc01g099190.2 | 17.7.1.2 | hormone metabolism.jasmonate.synthesis-degradation.lipoxygenase |
| mannan endo-1,4-beta-mannosidase 3 | Solyc12g013750.1 | 10.6.2 | cell wall.degradation.mannan-xylose-arabinose-fucose |
| pentatricopeptide repeat-containing protein At1g80270, mitochondrial (predicted) | Solyc12g020050.1 | 27.3.67 | RNA.regulation of transcription.putative transcription regulator |
| phosphomethylpyrimidine synthase, chloroplastic | Solyc06g006080.2 | 18.2, 29.2.2 | Co-factor and vitamine metabolism.thiamine; protein.synthesis.ribosome biogenesis |
| SBT1 protein precursor | Solyc04g078110.1 | 29.5.11.4.2, 30.3 | protein.degradation.ubiquitin.E3.RING; signalling.calcium |
| SKP1-like protein 21 isoform X1 (predicted) | Solyc06g036070.2 | 29.5.11.4.3.1 | protein.degradation.ubiquitin.E3.SCF.SKP |
| sucrose synthase 7-like | Solyc02g081300.2 | 2.2.1.5, 20.2.1 | major CHO metabolism.degradation.sucrose.Susy; stress.abiotic.heat |
| sufE-like protein 2, chloroplastic isoform X2 (predicted) | Solyc05g013710.2 | 16.2.1.10, 18.7, 29.4 | secondary metabolism.phenylpropanoids.lignin biosynthesis.CAD; Co-factor and vitamine metabolism.iron-sulphur clusters; protein.postranslational modification |
| zinc finger CCCH domain-containing protein 46-like isoform X2 (predicted) | Solyc01g008600.2 | 27.3.99, 27.4 | RNA.regulation of transcription.unclassified; RNA.RNA binding |

BIN codes and names adopted from http://www.gomapman.org/search/gmm

**Table S5.** **Description of the differentially abundant fruit proteins in (A) cv. Aragon, (B) cv. Gladis.**

A)

| Protein name | Gene name | Description | Functions | References |
| --- | --- | --- | --- | --- |
| 14-3-3 protein 2 | Solyc12g057110.2 | signal transduction (controlling metabolism, hormone signaling, cell division, responses to abiotic and biotic stimuli) | A significant overabundance of the 14-3-3 protein was found in the presence of As, while the addition of Si seemed to avoid the modulation of this protein. | [1, 2, 3, 4] |
| actin-51 (Fragment) | Solyc11g005330.1 | cytoskeleton component, cytoplasmic streaming, cell shape determination, cell division, organelle movement and extension growth | Our results showed a significant overabundance of the Actin-51 fragment only with the As treatment and a slight underabundance with As+Si. | [5, 6, 7] |
| ADP/ATP translocator | Solyc11g062130.1 | major carbohydrates metabolism; ADP/ATP carrier | The protein was missing in the As treatment, not modulated in the As+Si treatment. | [8] |
| alcohol dehydrogenase 2 | Solyc06g059740.2 | fermentation, interconversion of aldehyde and alcohol forms of flavor volatiles | We observed an overabundance of the protein in fruits treated with As, control and As+Si treatment had almost the same concentrations of this protein. | [9, 10] |
| annexin p34 | Solyc04g073990.2 | signalling, adaptation, abiotic stress response, membrane binding, binding to actin (exocytosis and signalling), peroxidase, ATP and GTP hydrolisis | We found a significant overabundance of annexin p34 in the presence of As, while the addition of As+Si did not cause annexin modulation. | [11, 12, 13, 14] |
| ATP-dependent Clp protease ATP-binding subunit clpA homolog CD4B, chloroplastic | Solyc12g042060.1 | protein degradation, ATP-dependent chaperones | An underabundance of the ATP-dependent Clp protease ATP-binding subunit was found with both treatments, but significantly higher with As. | [15] |
| calmodulin | Solyc03g098050.2 | signal transduction (cell division, cell elongation, ion transport, secondary metabolism, plant defense, abiotic stress, heavy metal toxicity) | A significant overabundance of calmodulin was found with both treatments. Moreover, the Ca^2+^-CaM signaling is involved in adaptation to heat stress-induced oxidative stress in *Solanum lycopersicum* L. leaves suggesting a similar behavior in adaptation to arsenic-induced oxidative stress in fruits. | [16, 17] |
| cytosolic sulfotransferase 12-like (predicted) | Solyc05g011870.1 | hormone metabolism, lipid metabolism, sulfoconjugation catalysis | We found a significant overabundance of the cytosolic sulfotransferase 12-like with both treatments, supporting the hypothesis proposed by Nguyen et al. [20] of an involvement of sulfate assimilation and GSH metabolism in the detoxification of As. | [18, 19, 20] |
| eukaryotic translation initiation factor 5A-2 | Solyc07g005560.2 | protein synthesis regulation, translation elongation, mRNA turnover and decay, cell proliferation, leaf and root growth, seed yield, leaf, flower and fruit senescence and programmed cell death, abiotic stress response | We found this protein only in the presence of As+Si, where it was significantly overabundant. | [21, 22, 23] |
| F-box/WD-40 repeat-containing protein At5g21040-like (predicted) | Solyc06g073650.1 | plant growth and development | We found overabundance of this F-box protein, with a significant difference between treatments since administration of As increased its abundance in respect to As accompanied with Si. | [24, 25] |
| growth-regulating factor 9-like (predicted) | Solyc08g079800.2 | transcription factor; member of 14-3-3 family, abiotic and biotic stress response | We observed a significant overabundance of GRF9 with both treatments, suggesting the necessity of an increased transcriptional activity in response to As stress. | [1, 2, 26, 27] |
| nucleolin 1 isoform X1 (predicted) | Solyc02g021220.1 | signal transduction (regulation of cell growth, DNA replication, plant growth and development); rRNA synthesis and ribosome biogenesis | In our study, we found an underabundance of nucleolin in the presence of As, suggesting an inhibition of fruit growth and development due to As toxicity. | [28] |
| pathogenesis-related protein STH-2-like | Solyc09g090980.2 | biotic and abiotic stress response | A significant underabundance of the protein was observed, induced by As, but the addition of As+Si did not modulate its abundance. | [29, 30] |
| PHD finger protein ALFIN-LIKE 2-like (predicted) | Solyc10g076690.1 | transcription factor, biotic and abiotic stress response | The protein was missing in untreated and As treated samples, while it was significantly overabundant in the presence of As+Si, suggesting the role of Si in its modulation. | [31, 32] |
| probable lipid-A-disaccharide synthase, mitochondrial (predicted) | Solyc07g049740.2 | signal transduction; lipid IV(A) biosynthesis | This protein showed a significant modulation between treatments, with a strong inhibition in the presence of As+Si in respect to the treatment only with As. | [33] |
| protein LURP-one-related 10-like (predicted) | Solyc10g085420.1 | signaling, gene regulation, phospholipid scramblase, membrane tethered transcription factors | In our experiments, there was a high level of LURP protein in As treatments, while a low level was found with As+Si, the difference between the treatments was significant. | [34, 35] |
| putative cyclin-D6-1 | Solyc07g054950.1 | cell division, cell expansion | The putative cyclin-D6-1 was missing in the presence of As and was not modulated in the presence of As+Si. | [7] |
| seed biotin-containing protein SBP65-like isoform X1 | Solyc10g008040.2 | biotin storage, properties similar to late embryogenesis abundant proteins (LEA) which serve to protect other proteins from cold and osmotic stress | A significant under regulation of SBP65 was found with both treatments but stronger in the presence of Si. | [36] |
| small heat shock protein, chloroplastic | Solyc03g082420.2 | abiotic stress response, embryogenesis, seed germination, fruit maturation | In our study the sHSP was significantly modulated only in the presence of As+Si, suggesting that Si was responsible for its overabundance. | [3, 29, 37, 38, 39] |
| uncharacterized protein LOC101252956 (predicted) | Solyc03g005820.2 | transport of cytokinin | In our study, this protein was present only in the non-treated samples, otherwise missing with both treatments. | [40] |
| uncharacterized protein LOC101257285 | Solyc02g078030.1 | DNA-binding WRKY transcription factors, plant growth and development, biotic and abiotic stress | It was found a significant overabundance of the protein in the presence of As, while it was not modulated with the addition of As+Si. | [41] |
| vacuolar H^+^-ATPase A2 subunit isoform | Solyc06g063330.2 | generation of proton gradients across the tonoplast and other compartments of the endomembrane systems, maintenance of cytoplasmic ion and pH homeostasis | We observed a significant underabundance of the protein only in the presence of As+Si, suggesting the predominant role of Si in its modulation | [26, 30, 42] |

| Protein name  B) | Gene name | Description | Comments | References |
| --- | --- | --- | --- | --- |
| 1-aminocyclopropane-1-carboxylate oxidase homolog isoform 1 | Solyc09g089580.2 | ethylene biosynthesis | In our work, we found a downregulation of the protein, with both treatments. | [43, 44] |
| 26S protease regulatory subunit 7 homolog A-like (predicted) | Solyc06g063140.2 | regulated degradation of polyubiquitinated proteins | In the presence of As the protein was missing in respect to control, the administration of As+Si yielded the same concentrations of this protein as in the control. | [45, 46, 47] |
| 40S ribosomal protein S3a (predicted) | Solyc06g072490.2 | structural constituent of ribosome; protein translation | We found it in untreated and As+Si treated samples, while it was missing in the presence of As. | [48] |
| ADP/ATP translocator | Solyc11g062130.1 | major carbohydrates metabolism; ADP/ATP carrier | It was significantly overabundant in the treatment with As, while As+Si did not modulate its abundance. | [8] |
| alcohol dehydrogenase 2 | Solyc06g059740.2 | fermentation, interconversion of aldehyde and alcohol forms of flavor volatiles | Here it was overabundant in As+Si, missing in As treatment. In cv. Aragon ADH2 was not modulated under As+Si treatment, overabundant with as treatment. | [9, 10] |
| aldo-keto reductase family 4 member C9 (predicted) | Solyc00g015750.1 | oxidoreduction of aldehyde and keto substrates | In our experiment, it was missing in As+Si in respect to control, slightly underabundant in As treatment. | [49, 50, 51] |
| argininosuccinate lyase | Solyc04g076320.2 | arginine biosynthetic process | In our case, the protein was underabundant for As treatment, but missing for As+Si in respect to control. | [52, 53, 54] |
| ATP synthase subunit beta, chloroplastic | Solyc01g007320.2 | ATP synthesis | We found an underabundance of this protein in both treatments. | [55, 56, 57] |
| calcium-dependent protein kinase | Solyc03g033540.2 | signaling, lipid metabolism, protein post-translational modification | The protein was missing in the untreated samples, while it was significantly overabundant with both treatments. | [58] |
| CBL-interacting protein kinase | Solyc05g052270.1 | tolerance acquisition and acclimation under environmental stresses | The kinase was missing in untreated and As treated samples while it was overabundant in the presence of As+Si. | [59] |
| cell division cycle protein 48 homolog (predicted) | Solyc06g074980.2 | cell wall biosynthesis, ubiquitination of misfolded proteins | In our case, the treatments modulated the abundance of the protein in an opposite manner, with a significant difference between them (over in As, under in As+Si). | [60] |
| cytochrome P450 85A1 | Solyc02g089160.2 | hormone metabolism | Our results showed an underabundance of the cytochrome P450 85A1 with both treatments, particularly significant with As+Si treatment. | [61] |
| dihydroflavonol-4-reductase | Solyc02g085020.2 | anthocyanin biosynthesis | We found that DFR was overabundant when As was administered, while underabundant when As+Si were added to the plants. | [62] |
| E3 ubiquitin-protein ligase CHIP (predicted) | Solyc06g083150.2 | co-chaperones of the heat shock cognate 70; protein ubiquitination and degradation; regulation of biological processes | It was found at very high levels in both treatments but it was missing in untreated samples. | [63, 64, 65] |
| enolase | Solyc09g009020.2 | glycolysis | In our experiment, the protein levels were modest under As treatment, not modulated during As+Si treatment. | [37, 66, 67, 68] |
| F-box protein At4g00755-like (predicted) | Solyc03g026170.2 | plant growth and development | The treatment with As caused an underabundance, while the treatment with As+Si did not change its level. | [24] |
| F-box/WD-40 repeat-containing protein At5g21040-like | Solyc06g073650.1 | plant growth and development | Both he treatments with As and as+Si caused an underabundance of the protein in respect to control | [24] |
| GDSL esterase/lipase EXL3-like (predicted) | Solyc04g082390.2 | hydrolysis and synthesis of ester compounds, biosynthesis of cutin | We observed an overabundance of the protein in the presence of As, in AS+Si treatments it was not modulated. | [37, 69, 70] |
| growth-regulating factor 9-like | Solyc08g079800.2 | transcription factor; member of 14-3-3 family, abiotic and biotic stress response | It was overabundant in the presence of As+Si, while it was missing in the As treatment in respect to control. | [1, 2, 26, 71] |
| heat shock cognate 70 kDa protein 2 | Solyc10g086410.2 | protein stabilization, folding, assembly and translocation | An overabundance of the protein was found under both treatments. | [72] |
| heavy metal-associated isoprenylated plant protein 26-like | Solyc01g111600.2 | metallochaperones | An overabundance of the protein with both treatments was observed, significantly higher with the addition of As+Si. | [73] |
| linoleate 9S-lipoxygenase B | Solyc01g099190.2 | oxylipin biosynthesis | The protein was significantly underabundant in the presence of As, while the addition of As+Si did not significantly modulate its abundance. | [58, 74, 75] |
| mannan endo-1,4-beta-mannosidase 3 | Solyc12g013750.1 | biosynthesis of mannans | In our experiment the protein was underabundant and its levels were lower in As+Si than is As treatments. | [76, 77] |
| pentatricopeptide repeat-containing protein At1g80270, mitochondrial (predicted) | Solyc12g020050.1 | influence on organelle biogenesis and function | The protein was detected only in the untreated samples, while it was missing in the treated ones. | [78] |
| phosphomethylpyrimidine synthase, chloroplastic | Solyc06g006080.2 | thiamine (vitamin B1) biosynthesis | In our case, the treatments modulated the abundance of the protein in an opposite manner: significant low abundance in As, significant high abundance in As+Si. | [79] |
| SBT1 protein precursor | Solyc04g078110.1 | selective protein degradation, plant-specific developmental processes, interactions with other organisms and cell death | It was found overabundant in both treatments while it was missing in the untreated samples. | [80] |
| SKP1-like protein 21 isoform X1 (predicted) | Solyc06g036070.2 | cell cycle regulation, signal transduction, transcription, defense responses | We found an overabundance of SKP1 protein with both treatments, confirming its involvement in abiotic stress response. | [81, 82] |
| sucrose synthase 7-like | Solyc02g081300.2 | energy metabolism, sugar import, cell wall synthesis and sink strength | We observed an underabundance of the protein with both treatments, higher with As+Si. | [83, 84] |
| sufE-like protein 2, chloroplastic isoform X2 (predicted) | Solyc05g013710.2 | electron transport, redox and non-redox catalysis, sensing of environmental stimuli, DNA repair, regulation of gene expression | We observed an opposite modulation induced by treatments, overabundance induced by As and underabundance by As+Si. | [85, 86] |
| zinc finger CCCH domain-containing protein 46-like isoform X2 (predicted) | Solyc01g008600.2 | RNA-binding proteins, regulation of mRNA processing, plant growth and development, fruit ripening, abiotic and biotic stresses | The protein was underabundant in both treatments, with a significant difference between them. | [87] |

Description of the differentially abundant fruit proteins in (A) cv. Aragon, (B) cv. Gladis, following a 14 day exposure to either the As or the As+Si treatment, numbers in the last column correspond to the relevant bibliography.

**References**

1. Lozano-Durán R, Robatzek S. 14-3-3 proteins in plant-pathogen interactions. Mol. Plant. Microbe. Interact. 2015;28:511–8.
2. Keller CK, Radwan O. The functional role of 14-3-3 proteins in plant-stress interactions. 2015;1.
3. Wang Y, Wang W, Cai J, Zhang Y, Qin G, Tian S. Tomato nuclear proteome reveals the involvement of specific E2 ubiquitin-conjugating enzymes in fruit ripening. Genome Biol. 2014;1–19.
4. Lu Y, Yasuda S, Li X, Fukao Y, Tohge T, Fernie AR, et al. Characterization of ubiquitin ligase SlATL31 and proteomic analysis of 14-3-3 targets in tomato fruit tissue (*Solanum lycopersicum* L.). J. Proteomics. 2016;143:254–64.
5. Liu P, Qi M, Xue X, Ren H. Dynamics and functions of the actin cytoskeleton during the plant cell cycle. Chinese Sci. Bull. 2011;56:3504–10.
6. Rodríguez-Celma J, Rellán-Álvarez R, Abadía A, Abadía J, López-Millán AF. Changes induced by two levels of cadmium toxicity in the 2-DE protein profile of tomato roots. J. Proteomics. 2010;73:1694–706.
7. Zhang T, Wang X, Lu Y, Cai X, Ye Z, Zhang J. Genome-wide analysis of the cyclin gene family in tomato. Int. J. Mol. Sci. 2013;15:120–40.
8. Haferkamp I, Fernie AR, Neuhaus HE. Adenine nucleotide transport in plants: much more than a mitochondrial issue. Trends Plant Sci. 2011;16:507–15.
9. Speirs J, Correll R, Cain P. Relationship between ADH activity, ripeness and softness in six tomato cultivars. Sci. Hortic. (Amsterdam). 2002;93:137–42.
10. Alexander L, Grierson D. Ethylene biosynthesis and action in tomato: a model for climacteric fruit ripening. J. Exp. Bot. 2002;53:2039–55.
11. Calvert CM, Gant SJ, Bowles DJ. Tomato annexins p34 and p35 bind to F-actin and display nucleotide phosphodiesterase activity inhibited by phospholipid binding. Plant Cell. 1996;8:333–42.
12. Hoshino D, Hayashi A, Temmei Y, Kanzawa N, Tsuchiya T. Biochemical and immunohistochemical characterization of Mimosa annexin. Planta. 2004;219:867–75.
13. Konopka-Postupolska D. Annexins: Putative linkers in dynamic membrane-cytoskeleton interactions in plant cells. Protoplasma. 2007;230:203–15.
14. Mortimer JC, Laohavisit A, Macpherson N, Webb A, Brownlee C, Battey NH, et al. Annexins: Multifunctional components of growth and adaptation. J. Exp. Bot. 2008;59:533–44.
15. K. Nishimura, K.J. van Wijk, Organization, function and substrates of the essential Clp protease system in plastids. Biochim Biophys Acta. 2015;1847:915–930
16. Tripathi BN. Stress Responses in Plants; Mechanisms of Toxicity and Tolerance. Springer Int. Publ. Switz. 2015.
17. Huang Y, Zhou Y, Wong H-C, Castiblanco A, Chen Y, Brown EM, et al. Calmodulin regulates Ca^2+^-sensing receptor-mediated Ca^2+^ signaling and its cell surface expression. J. Biol. Chem. 2010;285:35919–31.
18. Hirschmann F, Krause F, Papenbrock J. The multi-protein family of sulfotransferases in plants: composition, occurrence, substrate specificity, and functions. Front. Plant Sci. 2014;5:556.
19. Nimmagadda D, Cherala G, Ghatta S. Cytosolic sulfotransferases. Indian J. Exp. Biol. 2006;44:171–82.
20. Nguyen Q-TT, Huang T-L, Huang H-J. Identification of genes related to arsenic detoxification in Rice roots using microarray analysis. Int. J. Biosci. Biochem. Bioinforma. 2014;4:22–7.
21. Ma Y, Miura E, Ham BK, Cheng HW, Lee YJ, Lucas WJ. Pumpkin eIF5A isoforms interact with components of the translational machinery in the cucurbit sieve tube system. Plant J. 2010;64:536–50.
22. Xu J, Zhang B, Jiang C, Ming F. RceIF5A, encoding an eukaryotic translation initiation factor 5A in *Rosa chinensis*, can enhance thermotolerance, oxidative and osmotic stress resistance of Arabidopsis thaliana. Plant Mol. Biol. 2011;75:167–78.
23. Chou WC, Huang YW, Tsay WS, Chiang TY, Huang DD, Huang HJ. Expression of genes encoding the rice translation initiation factor, eIF5A, is involved in developmental and environmental responses. Physiol. Plant. 2004;121:50–7.
24. Stefanowicz K, Lannoo N, Van Damme EJM. Plant F-box proteins – Judges between life and death. CRC. Crit. Rev. Plant Sci. 2015;34:523–52.
25. Kuroda H, Yanagawa Y, Takahashi N, Horii Y, Matsui M. A comprehensive analysis of interaction and localization of Arabidopsis SKP1-LIKE (ASK) and F-Box (FBX) proteins. PLoS One. 2012;7.
26. Suzuki M, Takahashi S, Kondo T, Dohra H, Ito Y, Kiriiwa Y, et al. Plastid proteomic analysis in tomato fruit development. PLoS One. 2015;10:1–25.
27. He Y, Wu J, Lv B, Li J, Gao Z, Xu W, et al. Involvement of 14-3-3 protein GRF9 in root growth and response under polyethylene glycol-induced water stress. J. Exp. Bot. 2015;66:2271–81.
28. Tajrishi MM, Tuteja R, Tuteja N. Nucleolin: The most abundant multifunctional phosphoprotein of nucleolus. Commun. Integr. Biol. 2011;4:267–75.
29. Pan X, Zhu B, Zhu H, Chen Y, Tian H, Luo Y, et al. iTRAQ Protein profile analysis of tomato green-ripe mutant reveals new aspects critical for fruit ripening. J. Proteome Res. 2014;13:1979–93.
30. Xu P, Jiang L, Wu J, Li W, Fan S, Zhang S. Isolation and characterization of a pathogenesis-related protein 10 gene (GmPR10) with induced expression in soybean (*Glycine max*) during infection with Phytophthora sojae. Mol. Biol. Rep. 2014;41:4899–909.
31. Kayum MA, Park J-I, Ahmed NU, Saha G, Chung M-Y, Kang J-G, et al. Alfin-like transcription factor family: characterization and expression profiling against stresses in *Brassica oleracea*. Acta Physiol. Plant. 2016;38:127.
32. Wei W, Zhang YQ, Tao JJ, Chen HW, Li QT, Zhang WK, et al. The Alfin-like homeodomain finger protein AL5 suppresses multiple negative factors to confer abiotic stress tolerance in Arabidopsis. Plant J. 2015;81:871–83.
33. Li C, Guan Z, Liu D, Raetz CRH. Pathway for lipid A biosynthesis in *Arabidopsis thaliana* resembling that of Escherichia coli. Proc. Natl. Acad. Sci. U. S. A. 2011;108:11387–92.
34. Knoth C, Eulgem T. The oomycete response gene LURP1 is required for defense against Hyaloperonospora parasitica in *Arabidopsis thaliana*. Plant J. 2008;55:53–64.
35. Bateman A, Finn RD, Sims PJ, Wiedmer T, Biegert A, Söding J. Phospholipid scramblases and Tubby-like proteins belong to a new superfamily of membrane tethered transcription factors. Bioinformatics. 2009;25:159–62.
36. Okekeogbu I, Ye Z, Sangireddy S, Li H, Bhatti S, Hui D, et al. Effect of Aluminum Treatment on Proteomes of Radicles of Seeds Derived from Al-Treated Tomato Plants. Proteomes. 2014;2:169–90.
37. Barsan C, Zouine M, Maza E, Bian W, Egea I, Rossignol M, et al. Proteomic Analysis of Chloroplast-to-Chromoplast Transition in Tomato Reveals Metabolic Shifts Coupled with Disrupted Thylakoid Biogenesis Machinery and Elevated Energy-Production Components. Plant Physiol. 2012;160:708–25.
38. Sun W, Van Montagu M, Verbruggen N. Small heat shock proteins and stress tolerance in plants. Biochim. Biophys. Acta - Gene Struct. Expr. 2002;1577:1–9.
39. Volkov RA, Panchuk II, Schöffl F. Small heat shock proteins are differentially regulated during pollen development and following heat stress in tobacco. Plant Mol. Biol. 2005;57:487–502.
40. Zwack PJ, Rashotte AM. Interactions between cytokinin signalling and abiotic stress responses. J. Exp. Bot. 2015;66:4863–71.
41. Rushton PJ, Somssich IE, Ringler P, Shen QJ. WRKY transcription factors. Trends Plant Sci. 2010;15:247–58.
42. Bageshwar UK, Taneja-Bageshwar S, Moharram HM, Binzel ML. Two isoforms of the A subunit of the vacuolar H+-ATPase in *Lycopersicon esculentum*: Highly similar proteins but divergent patterns of tissue localization. Planta. 2005;220:632–43.
43. Booker MA, DeLong A. Producing the ethylene signal: regulation and diversification of ethylene biosynthetic enzymes. Plant Physiol. 2015;169:42–50.
44. Barry CS, Llop-Tous MI, Grierson D. The regulation of 1-aminocyclopropane-1-carboxylic acid synthase gene expression during the transition from system-1 to system-2 ethylene synthesis in tomato. Plant Physiol. 2000;123:979–86.
45. Stone SL, Callis J. Ubiquitin ligases mediate growth and development by promoting protein death. Curr. Opin. Plant Biol. 2007;10:624–32.
46. Sako K, Yanagawa Y, Kanai T, Sato T, Seki M, Fujiwara M, et al. Proteomic analysis of the 26S proteasome reveals its direct interaction with transit peptides of plastid protein precursors for their degradation. J. Proteome Res. 2014;13:3223–30.
47. Moshe A, Gorovits R, Liu Y, Czosnek H. Tomato plant cell death induced by inhibition of HSP90 is alleviated by Tomato yellow leaf curl virus infection. Mol. Plant Pathol. 2016;17:247-260
48. Carroll AJ, Heazlewood JL, Ito J, Millar AH. Analysis of the Arabidopsis cytosolic ribosome proteome provides detailed insights into its components and their post-translational modification. Mol. Cell. Proteomics. 2008;7:347–69.
49. Agius F, González-Lamothe R, Caballero JL, Muñoz-Blanco J, Botella MA, Valpuesta V. Engineering increased vitamin C levels in plants by overexpression of a D-galacturonic acid reductase. Nat. Biotechnol. 2003;21:177–81.
50. Valpuesta V, Botella MA. Biosynthesis of L-ascorbic acid in plants: New pathways for an old antioxidant. Trends Plant Sci. 2004;9:573–7.
51. Sengupta D, Naik D, Reddy AR. Plant aldo-keto reductases (AKRs) as multi-tasking soldiers involved in diverse plant metabolic processes and stress defense: A structure-function update. J. Plant Physiol. 2015;179:40–55.
52. Tiburcio AF, Altabella T, Bitrián M, Alcázar R. The roles of polyamines during the lifespan of plants: From development to stress. Planta. 2014;240:1–18.
53. Jeandroz S, Wipf D, Stuehr DJ, Lamattina L, Melkonian M, Tian Z, et al. Occurrence , structure , and evolution of nitric oxide synthase – like proteins in the plant kingdom. Sci. Signal. 2016;9:1–9.
54. Zhang X, Ji N, Zhen F, Ren P, Li F. Metabolism of endogenous arginine in tomato fruit harvested at different ripening stages. Sci. Hortic. (Amsterdam). 2014;179:349–55.
55. Kohzuma K, Dal Bosco C, Meurer J, Kramer DM. Light- and metabolism-related regulation of the chloroplast ATP synthase has distinct mechanisms and functions. J. Biol. Chem. 2013;288:13156–63.
56. Chen X, Zhang W, Zhang B, Zhou J, Wang Y, Yang Q, et al. Phosphoproteins regulated by heat stress in rice leaves. Proteome Sci. 2011;9:37.
57. Chevalier F, Rossignol M. Proteomic analysis of *Arabidopsis thaliana* ecotypes with contrasted root architecture in response to phosphate deficiency. J. Plant Physiol. 2011;168:1885–90.
58. Wang L, Baldwin EA, Bai J. Recent Advance in Aromatic Volatile Research in Tomato Fruit: The Metabolisms and Regulations. Food Bioprocess Technol. 2016;9:203–16.
59. Yuasa T. A Flower Specific Calcineurin B-Like Molecule (CBL)-Interacting Protein Kinase (CIPK) Homolog in Tomato Cultivar Micro-Tom (*Solanum lycopersicum* L.). Am. J. Plant Sci. 2012;3:753–63.
60. Paul P, Simm S, Blaumeiser A, Scharf K-D, Fragkostefanakis S, Mirus O, et al. The protein translocation systems in plants - composition and variability on the example of *Solanum lycopersicum*. BMC Genomics. 2013;14:189.
61. Choudhary SP, Yu JQ, Yamaguchi-Shinozaki K, Shinozaki K, Tran LSP. Benefits of brassinosteroid crosstalk. Trends Plant Sci. 2012;17:594–605.
62. Wang H, Fan W, Li H, Yang J, Huang J, Zhang P. Functional characterization of dihydroflavonol-4-reductase in anthocyanin biosynthesis of purple sweet potato underlies the direct evidence of anthocyanins function against abiotic stresses. PLoS One. 2013;8:1–14.
63. Smith MC, Scaglione KM, Assimon VA, Patury S, Thompson AD, Dickey CA, et al. The E3 ubiquitin ligase CHIP and the molecular chaperone Hsc70 form a dynamic, tethered complex. Biochemistry. 2013;52:5354–64.
64. Stone SL. The role of ubiquitin and the 26S proteasome in plant abiotic stress signaling. Front. Plant Sci. 2014;5:135.
65. Mazzucotelli E, Belloni S, Marone D, De Leonardis A, Guerra D, Di Fonzo N, et al. The e3 ubiquitin ligase gene family in plants: regulation by degradation. Curr. Genomics. 2006;7:509–22.
66. Van der Streaten, Rodrigues-Pousada RA, Goodman HM, Van Montagu M. Plant enolase: gene structure, expression, and evolution. Plant Cell. 1991;3:719–35.
67. Manaa A, Faurobert M, Valot B, Bouchet J-P, Grasselly D, Causse M, et al. Effect of salinity and calcium on tomato fruit proteome. OMICS 2013;17:338–52.
68. Obiadalla-Ali H, Fernie AR, Kossmann J, Lloyd JR. Developmental analysis of carbohydrate metabolism in tomato (*Lycopersicon esculentum* cv. Micro-Tom) fruits. Physiol Plant 2004;120:196–204.
69. Girard a.-L, Mounet F, Lemaire-Chamley M, Gaillard C, Elmorjani K, Vivancos J, et al. Tomato GDSL1 is required for cutin deposition in the fruit cuticle. Plant Cell. 2012;24:3119–34.
70. T.H. Yeats, L.B.B. Martin, H.M. Viart, T. Isaacson, L. Zhao, A.J. Matas, G.J. Buda, D.S. Domozych, H. Mads, J.K.C. Rose, The identification of cutin synthase: formation of the plant polyester cutin, Nat Chem Biol. 8 (2012) 609–611. doi:10.1038/nchembio.960.
71. He Y, Wu J, Lv B, Li J, Gao Z, Xu W, et al. Involvement of 14-3-3 protein GRF9 in root growth and response under polyethylene glycol-induced water stress. J. Exp. Bot. 2015;66:2271–81.
72. Rocco M, D’Ambrosio C, Arena S, Faurobert M, Scaloni A, Marra M. Proteomic analysis of tomato fruits from two ecotypes during ripening. Proteomics. 2006;6:3781–91.
73. De Abreu-Neto JB, Turchetto-Zolet AC, De Oliveira LFV, Bodanese Zanettini MH, Margis-Pinheiro M. Heavy metal-associated isoprenylated plant protein (HIPP): Characterization of a family of proteins exclusive to plants. FEBS J. 2013;280:1604–16.
74. Howe GA, Schilmiller AL. Oxylipin metabolism in response to stress. Curr. Opin. Plant Biol. 2002;5:230–6.
75. Shen J, Tieman D, Jones JB, Taylor MG, Schmelz E, Huffaker A, et al. A 13-lipoxygenase, TomloxC, is essential for synthesis of C5 flavour volatiles in tomato. J. Exp. Bot. 2014;65:419–28.
76. Songsiriritthigul C, Buranabanyat B, Haltrich D, Yamabhai M. Efficient recombinant expression and secretion of a thermostable GH26 mannan endo-1,4-beta-mannosidase from Bacillus licheniformis in Escherichia coli. Microb. Cell Fact. 2010;9:20.
77. Prakash R, Johnston SL, Boldingh HL, Redgwell RJ, Atkinson RG, Melton LD, et al. Mannans in tomato fruit are not depolymerized during ripening despite the presence of endo-β-mannanase. J. Plant Physiol. 2012;169:1125–33.
78. Barkan A, Small I. Pentatricopeptide repeat proteins in plants. Annu. Rev. Plant Biol. 2014;65:415–42.
79. Rapala-Kozik M, Wolak N, Kujda M, Banas AK. The upregulation of thiamine (vitamin B1) biosynthesis in Arabidopsis thaliana seedlings under salt and osmotic stress conditions is mediated by abscisic acid at the early stages of this stress response. BMC Plant Biol. 2012;12:2.
80. Schaller A, Stintzi A, Graff L. Subtilases - versatile tools for protein turnover, plant development, and interactions with the environment. Physiol. Plant. 2012;145:52–66.
81. D. Zhao, N. Weimin, F. Baomin, H. Tianfu, Members of the Arabidopsis-SKP1-like gene family exhibit a variety of expression patterns and may play diverse roles in Arabidopsis, Mol. Plant-Microbe Interact. 16 (2003) 295–305. doi:10.1104/pp.103.024703.to.
82. Zhang Y, Wang C, Lin Q, Gao F, Ma Y, Zhang M, et al. Genome-wide analysis of phylogeny, expression profile and sub-cellular localization of SKP1-Like genes in wild tomato. Plant Sci. 2015;238:105–14.
83. Komatsu A, Moriguchi T, Koyama K, Omura M, Akihama T. Analysis of sucrose synthase genes in citrus suggests different roles and phylogenetic relationships. J. Exp. Bot. 2002;53:61–71.
84. Upadhyaya H, Shome S, Roy D, Bhattacharya MK. Arsenic induced changes in growth and physiological responses in *Vigna radiata* Seedling : effect of curcumin interaction. 2014;3609–18.
85. Narayana Murthy UM, Ollagnier-de-Choudens S, Sanakis Y, Abdel-Ghany SE, Rousset C, Ye H, et al. Characterization of *Arabidopsis thaliana* SufE2 and SufE3: functions in chloroplast iron-sulfur cluster assembly and NAD synthesis. J. Biol. Chem. 2007;282:18254–64.
86. Couturier J, Touraine B, Briat J-F, Gaymard F, Rouhier N. The iron-sulfur cluster assembly machineries in plants: current knowledge and open questions. Front. Plant Sci. 2013;4:259.
87. Peng X, Zhao Y, Cao J, Zhang W, Jiang H, Li X, et al. CCCH-type zinc finger family in maize: Genome-wide identification, classification and expression profiling under abscisic acid and drought treatments. PLoS One. 2012;7.
