# Supplementary material for: Target proteins reprogrammed by As and As + Si treatments in Solanum lycopersicum L. fruit
Source: BMC Plant Biol. 2017 Nov 21;17:210. doi: 10.1186/s12870-017-1168-2 (PMC5696772; doi:10.1186/s12870-017-1168-2)
Supplement: Supplementary file 5 — 2D gel electrophoresis images of fruit proteins from A) Aragon, and B) Gladis. (PDF 973 kb) [file 12870_2017_1168_MOESM5_ESM.pdf]

Figure S2: 2D gel electrophoresis images of fruit proteins from A) Aragon, and B) Gladis.

A)

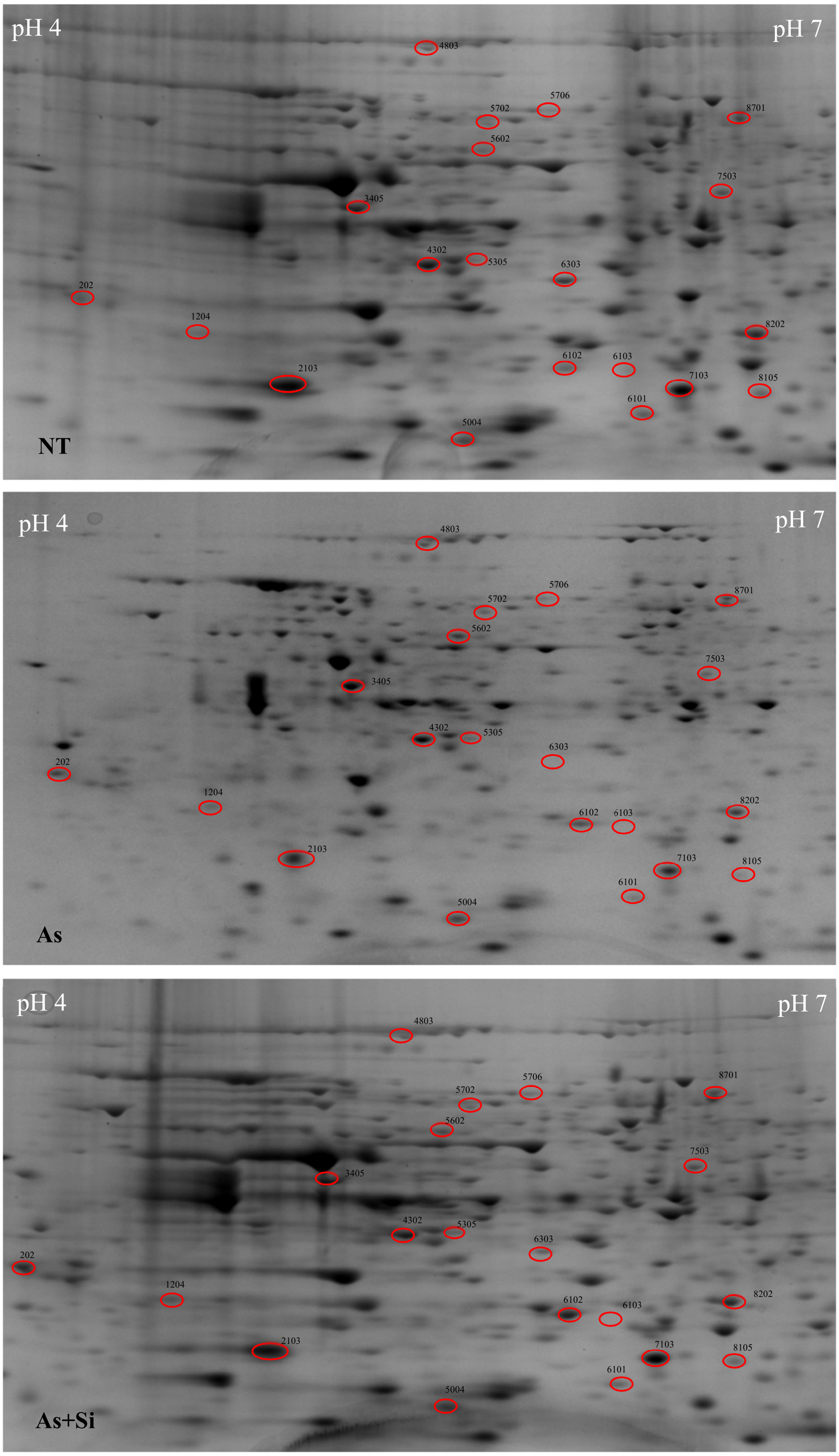

B)

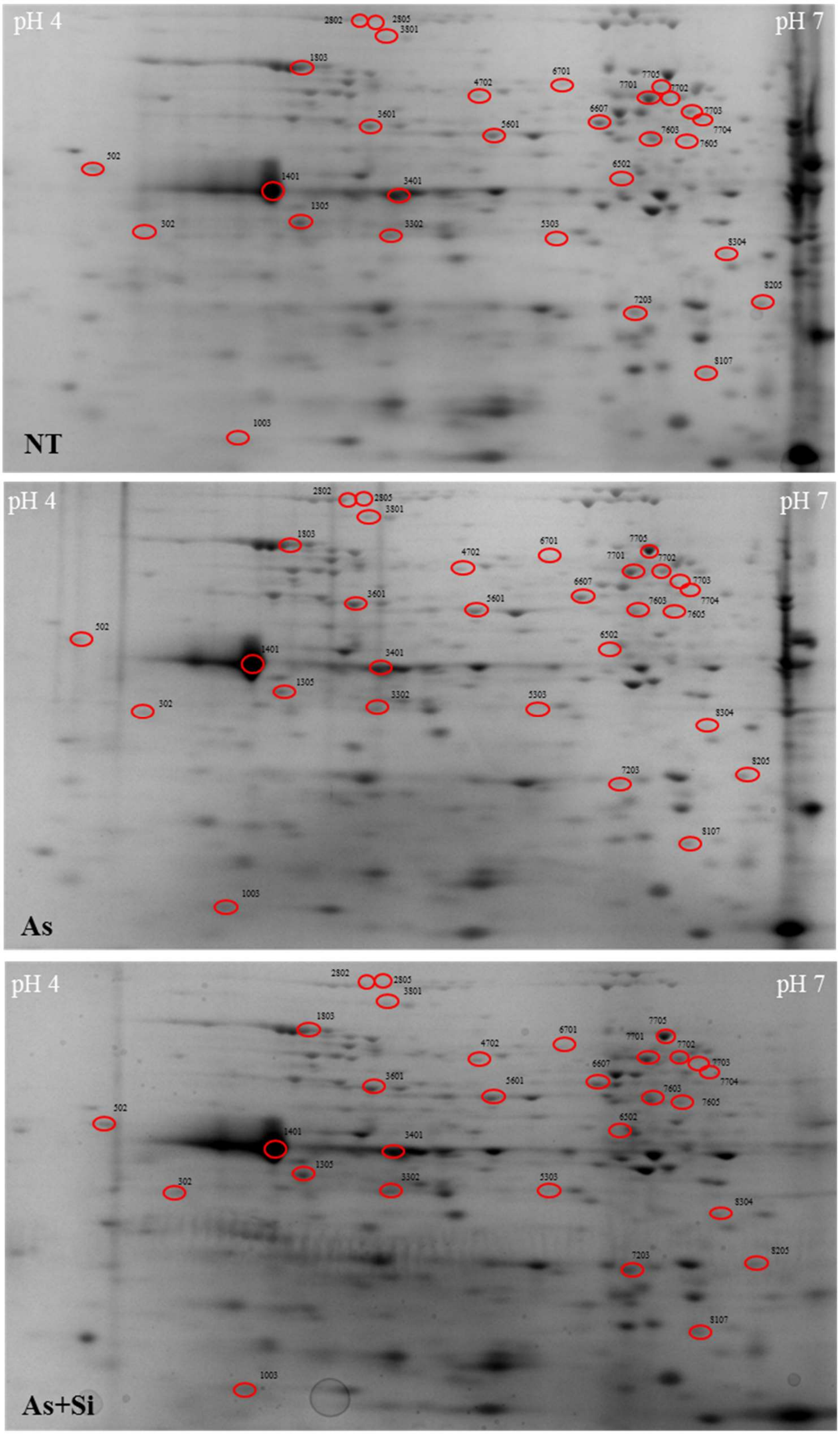

Red circles indicate the features selected on the basis of differential intensity, and are numbered as in Table S1.
