# Supplementary material for: Target proteins reprogrammed by As and As + Si treatments in Solanum lycopersicum L. fruit
Source: BMC Plant Biol. 2017 Nov 21;17:210. doi: 10.1186/s12870-017-1168-2 (PMC5696772; doi:10.1186/s12870-017-1168-2)
Supplement: Supplementary file 6 — Heat map of the differentially abundant proteins present in A) Aragon, B) Gladis. (PDF 737 kb) [file 12870_2017_1168_MOESM6_ESM.pdf]

Figure S3. Heat map of the differentially abundant proteins present in A) Aragon, B) Gladis.

A)

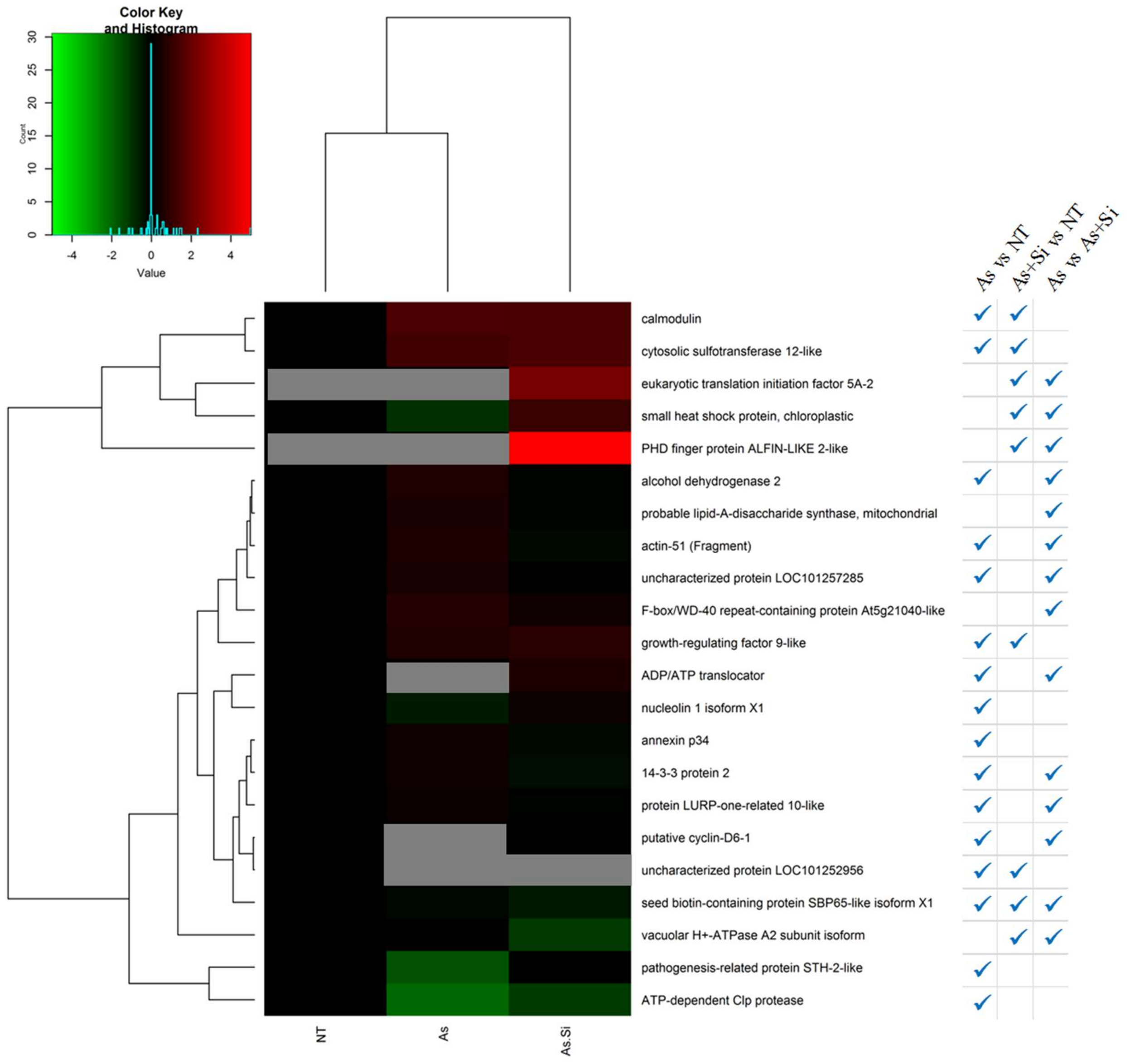

B)

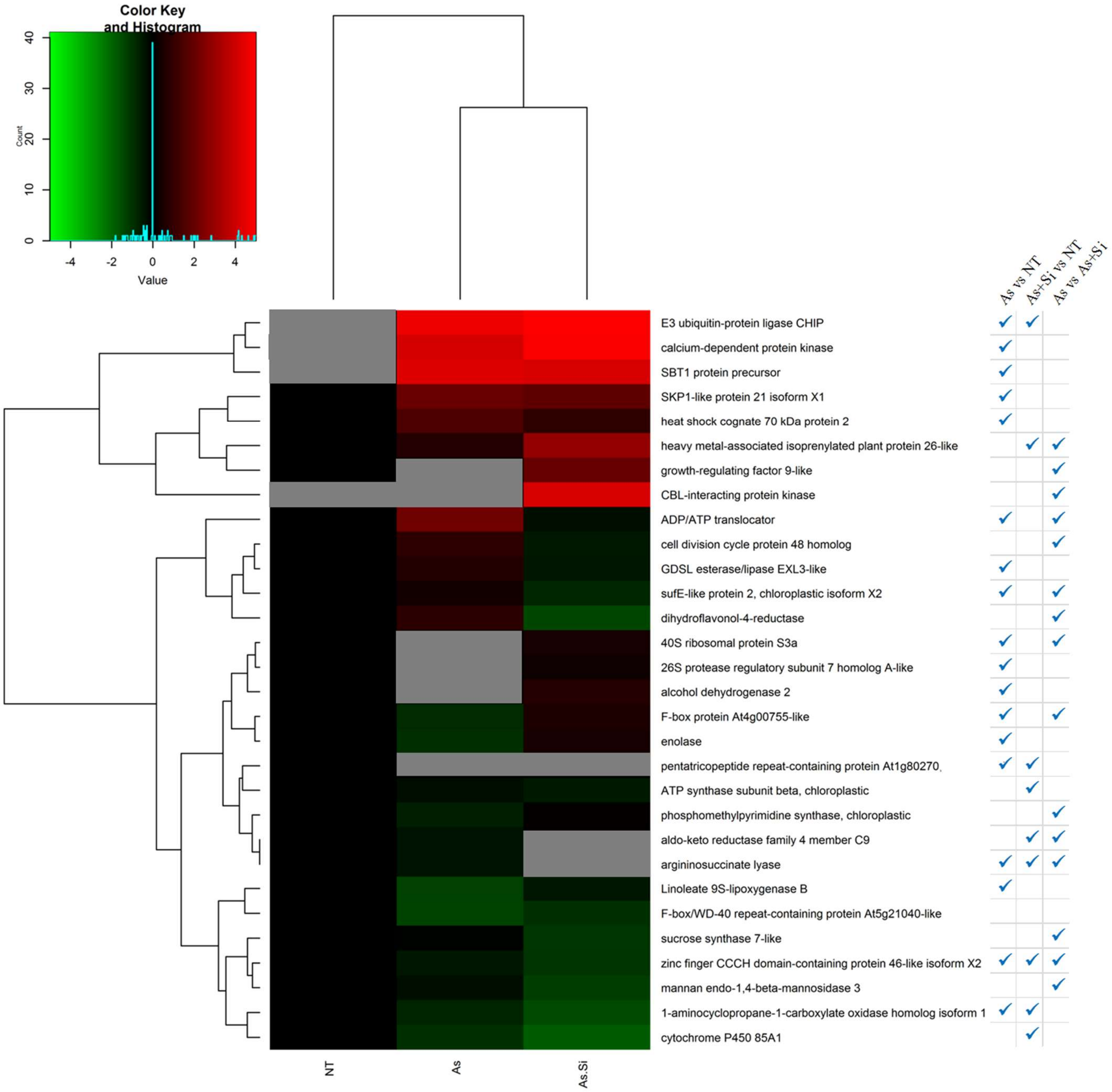

Proteins abundance variation is reported for comparisons between treated and non- treated (nt) plants. *Red*: over-abundant proteins in the treatment compared to nt; *green*: under-abundant proteins in the treatment compared to nt; *black*: no difference in abundance between the treatment and nt; *grey*: protein absent.
