# Supplementary material for: Target proteins reprogrammed by As and As + Si treatments in Solanum lycopersicum L. fruit
Source: BMC Plant Biol. 2017 Nov 21;17:210. doi: 10.1186/s12870-017-1168-2 (PMC5696772; doi:10.1186/s12870-017-1168-2)
Supplement: Supplementary file 7 — Representation of the differentially Differentially abundant fruit proteins involved in the “Biotic Stress” response MapMan pathway. (PDF 578 kb) [file 12870_2017_1168_MOESM7_ESM.pdf]

Figure S4. **Differentially abundant fruit proteins involved in the "Biotic Stress" response MapMan pathway**.

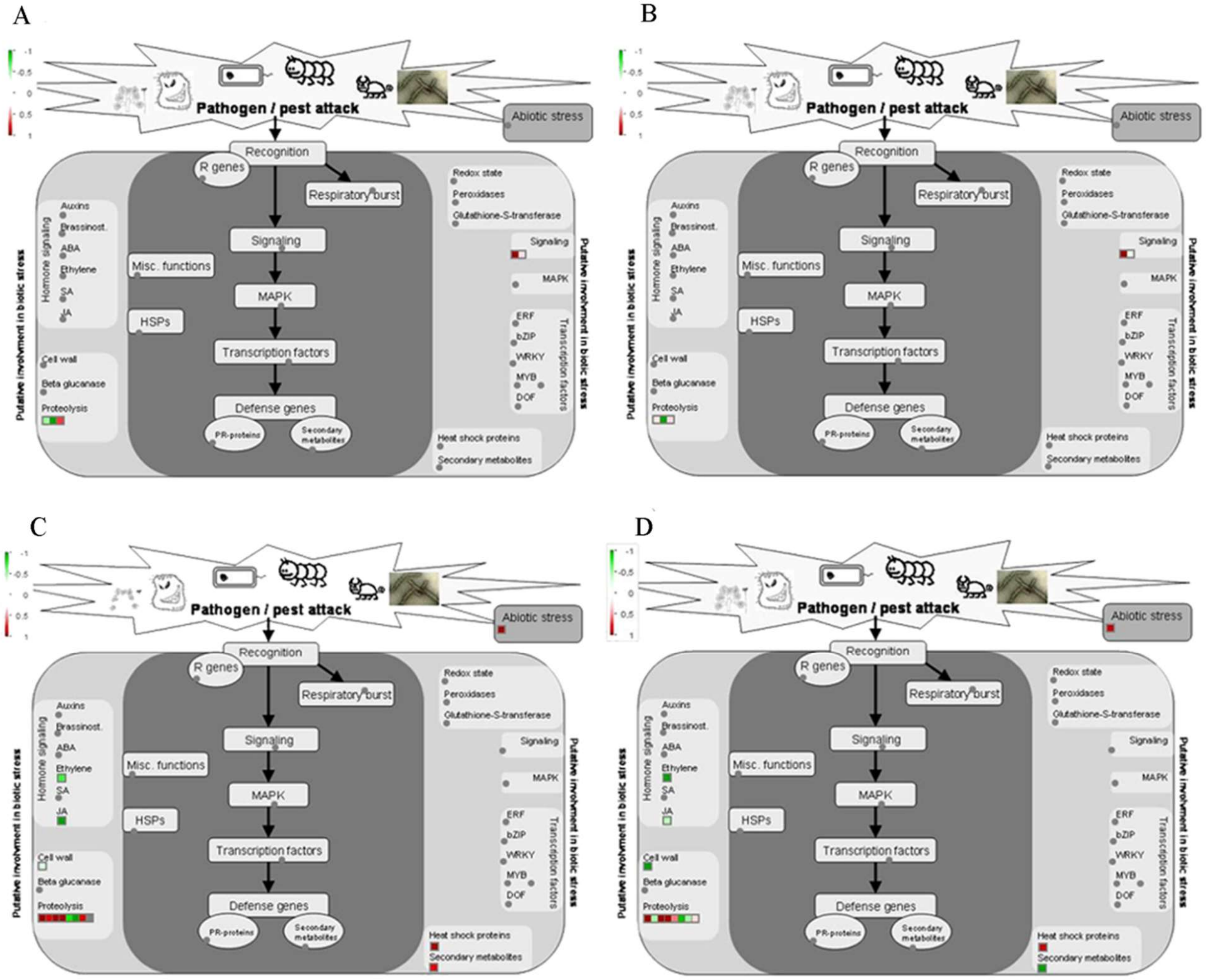

A, C: response to the As treatment; B, D response to the As+Si treatment, for (A, B) cv. Aragon and (C, D) cv. Gladis. Proteins more and less abundant in the treated compared to the non-treated plants are shown in shades of, respectively, *red* and *green*. Absent proteins are represented in *grey*.
