# Supplementary material for: Target proteins reprogrammed by As and As + Si treatments in Solanum lycopersicum L. fruit
Source: BMC Plant Biol. 2017 Nov 21;17:210. doi: 10.1186/s12870-017-1168-2 (PMC5696772; doi:10.1186/s12870-017-1168-2)
Supplement: Supplementary file 8 — Representation of the dDifferentially abundant fruit proteins in Gladis involved in the “Ubiquitin Dependent Degradation” MapMan. (PDF 444 kb) [file 12870_2017_1168_MOESM8_ESM.pdf]

Figure S5. Differentially abundant fruit proteins in Gladis involved in the "Ubiquitin Dependent Degradation" MapMan.

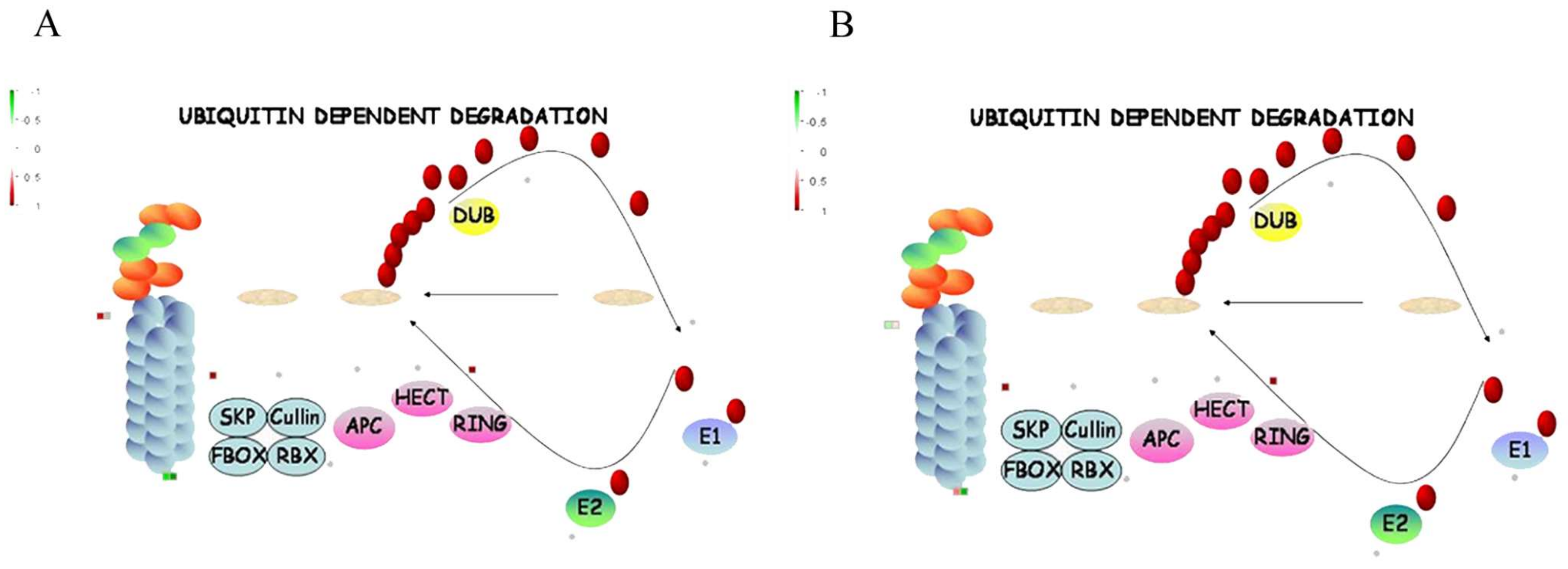

Response to A) As or B) As+Si treatments. Proteins more and less abundant in the treated compared to the non-treated plants are shown in shades of, respectively, *red* and *green*. Absent proteins are represented in *grey*.
